# Supplementary material for: Nitrous oxide respiration in acidophilic methanotrophs
Source: Nat Commun. 2024 May 18;15:4226. doi: 10.1038/s41467-024-48161-z (PMC11102522; doi:10.1038/s41467-024-48161-z)
Supplement: Supplementary file 1 — Supplementary Information [file 41467_2024_48161_MOESM1_ESM.pdf]

Supplementary Information for

Nitrous oxide respiration in acidophilic methanotrophs

Samuel Imisi Awala[a,b,1], Joo-Han Gwak[a,1], Yongman Kim[a], Man-Young Jung[c,d,e], Peter F. Dunfield[f], Michael Wagner[g,h], and Sung-Keun Rhee[a,*]

[a]Department of Biological Sciences and Biotechnology, Chungbuk National University, 1 Chungdae-ro, Seowon-Gu, Cheongju 28644, Republic of Korea

[b]Center for Ecology and Environmental Toxicology, Chungbuk National University, 1 Chungdae-Ro, Seowon-Gu, Cheongju, 28644, South Korea

[c]Interdisciplinary Graduate Programme in Advance Convergence Technology and Science, Jeju National University, Jeju, Republic of Korea

[d]Department of Science Education, Jeju National University, Jeju, Republic of Korea

[e]Jeju Microbiome Center, Jeju National University, Jeju, Republic of Korea

[f]Department of Biological Sciences, University of Calgary, 2500 University Dr. NW, Calgary, AB T2N 1N4, Canada

[g]Centre for Microbiology and Environmental Systems Science, Department of Microbiology and Ecosystem Science, Division of Microbial Ecology, University of Vienna, Althanstrasse 14, A-1090 Vienna, Austria

[h]Center for Microbial Communities, Department of Chemistry and Bioscience, Aalborg University, Fredrik Bajers Vej 7H, 9220 Aalborg, Denmark

[1] S.I.A. and J-H.G. contributed equally to this work.

*Sung-Keun Rhee

Email: rhees@chungbuk.ac.kr **Phone**: +82-43-261-2300. Fax: 82-43-264-9600.

The file includes:

Supplementary Figures and Tables

Supplementary Notes
Supplementary Figures 1 to 12
Supplementary References

Supplementary Notes

Supplementary Note 1
Conservation of metal-binding residues in N_2OR of methanotrophs

The nine histidine residues of the beta-propeller domain implicated in Cu_Z-binding were completely conserved (Supplementary Fig. 2). Like the clade I NosZ proteins, the Cu_Z-binding motifs of the enzymes encoded in *Methylocystis* and *Methylocella* strains associated with the first two histidine (DxHHxH) and the last histidine (EPHD), were also completely conserved. These residues were less completely conserved in strain IT6 (i.e., DxHH and EPH) as seen in other clade II NosZ proteins. Considering the extremely acidophilic and thermophilic nature of strain IT6, the conservation of the key residues is consistent with its function as a NosZ protein.

Supplementary Note 2
N_2O reduction kinetics

N_2O depletion in strains T4 and IT6 followed Michaelis-Menten kinetics (Supplementary Fig. S7). We could obtain a maximum N_2O reduction rate ($V_{max(app)}$) of 1.122 ± 0.005 mmol $N_2O \cdot h^{-1} \cdot g\ DW^{-1}$ and an apparent N_2O affinity ($K_{m(app)}$) of 5.937 ± 0.005 µM for the clade II N_2O-reducer, strain T4. The nitrous oxide reductase kinetics of strain IT6 cells showed a lower N_2O reduction rate ($V_{max(app)}$ = 0.414 ± 0.003 mmol $N_2O \cdot h^{-1} \cdot g\ DW^{-1}$) but a higher affinity constant value ($K_{m(app)}$) = 1.128 ± 0.043 µM N_2O). The affinity constant values of strain T4 and strain IT6 were consistent with those of clade I and clade II N_2O-reducers[1, 2], respectively.

Supplementary Note 3
Transcriptomics

Other denitrification genes (O_2 replete vs. anoxic/suboxic conditions)**.** Interestingly, even though N_2O was the only electron acceptor present and NH_4^+ was the only nitrogen source provided, other denitrification enzyme genes were also upregulated (Fig. 6, Supplementary Fig. 9, Supplementary Dataset 5) in *Methylocella tundrae* T4 cells grown on CH_4 and CH_3OH in response to N_2O addition in suboxic and anoxic conditions, respectively. The expression of genes encoding respiratory NAR (*narGHJI*; 4–102-fold upregulation) and NOR (*norBC*; 2.6–58-fold upregulation) in strain T4 cells grown on CH_4 and CH_3OH was greatly increased in anoxic and suboxic N_2O-respiring conditions. Similarly, in *Methylacidiphilum caldifontis* IT6, a gene encoding NIR (*nirK*; IT6_01798) and genes encoding NOR (*norBC*; IT6_01320–1) were about 2-fold and 3.5-fold upregulated in the N_2O-respiring anoxic CH_3OH + N_2O conditions, respectively, compared to the O_2-replete CH_3OH + O_2 conditions (Supplementary Fig. 10, Supplementary Dataset 7) in the absence of NO_3^- or NO_2^- in the growth medium. Low oxygen and the presence of NO_3^- and NO_2^- as terminal electron acceptors are necessary for the expression of the genes of the denitrification pathway in other bacteria[3-5]. Since neither NO_3^- nor NO_2^- were added to the cultures, the upregulation of NAR genes in strain T4 was probably influenced by the low O_2 concentrations and/or presence of N_2O. Although there is little evidence to support the upregulation of denitrification genes by N_2O, in *Wolinella succinogenes*, an N_2O reducer, the periplasmic nitrate reductase (Nap), cytochrome *c* nitrite reductase (Nrf), and N_2O reductase (Nos) were upregulated in response to the presence of N_2O (ref.[6]). In this strain, N_2O likely induced the Nap and Nrf systems independently even without notable amounts of NO_3^- or NO (ref.[6]). Also, exposure of *Paracoccus denitrificans* cells to N_2O under anoxia promoted NirS expression and synthesis[7]. Significant upregulation of genes encoding NOR (Supplementary Dataset 5) and NOR regulatory proteins in strain T4 might be related to RNS detoxification (see Main manuscript: *Nitrate and nitrite reduction in* Methylocella *species*).

Expression of denitrification transcriptional regulators. Regulatory proteins like the CRP/FNR family of transcriptional regulators, including FnrP (fumarate and nitrate reduction protein) and NNR (nitrite reductase and nitric oxide reductase regulator), respond to environmental signals like O_2 and NO to regulate the transcription and expression of denitrification genes[8, 9]. FnrP contains an oxygen-sensitive [4Fe-4S] cluster and regulates the oxygen-dependent transcriptional activation of many genes, including the *nar* operon[8]. Upstream of the *nos* genes of strain T4, we found three genes whose expression was upregulated (~2–29-fold) in suboxic CH_4 + O_2 + N_2O and anoxic CH_3OH + N_2O conditions (Supplementary Dataset 5). These include T4_03938, which encodes the NNR; T4_03939, which encodes the FnrP; and T4_03940, which encodes the flavodiiron protein rubrerythrin involved in the reduction of O_2 and/or NO to H_2O and N_2O (ref.[10]). Two genes encoding the FnrP (T4_02964 and T4_02965), that activate NAR genes expression in response to O_2 deprivation[11] were found immediately downstream of the NAR genes, and their expressions were upregulated (~ 2–27-fold) in response to N_2O respiration in suboxic and anoxic conditions (Supplementary Dataset 5). The expression of genes encoding NOR regulatory proteins (T4_00479–80), FnrP, and NNR, which are located immediately downstream of the *nor* genes, was also found to be significantly upregulated (14- to 21-fold) in the suboxic CH_4 + O_2 + N_2O growth conditions (Supplementary Dataset 5). This suggests that the FnrP- and NNR-encoding genes are involved in upregulations of strain T4 denitrification genes in response to O_2 limitation as observed in *Paracoccus denitrificans*[11]. Overall, data from the transcriptome analysis reveals that the expression of denitrification genes in *Methylocella tundrae* T4 is regulated during N_2O respiration in suboxic or anoxic conditions. The ability of strain T4 to upregulate the expression of denitrification genes in suboxia correlates well with its ability to employ a hybrid respiration system (i.e., simultaneous respiration of O_2 and N_2O) during CH_4 oxidation.

Terminal oxidase. Three types of aerobic terminal oxidases (TOs) are encoded in the genome of strain T4, and their gene expression pattern is presented in Supplementary Datasets 5 and 6. Among these are one *cbb*$_3$ (high-affinity TO), two *bd* (high-affinity TO), and two *aa*$_3$ (low-affinity TO)[12]. In the case of strain IT6, three types of TOs are encoded in its genome (Supplementary Dataset 7), as in most verrucomicrobial methanotrophs[13]. They include one *cbb*$_3$ (high-affinity TO), two *ba*$_3$ (medium-affinity TO), and two *aa*$_3$ (low-affinity TO)[12, 14]. Due to the presence of multiple types of TOs, both strains T4 and T6 are well-suited to environments with fluctuating oxygen concentrations including peatlands, paddy soils, sulfidic oxygen-limited volcanic and geothermal ecosystems.

Strain T4 cells oxidizing methanol and respiring N_2O in anoxic CH_3OH + N_2O conditions expressed all the genes encoding all terminal oxidases. Of these TOs, expression of genes encoding two of its high-affinity TOs, *bd*-type oxidase (T4_00262–6) and *cbb*$_3$-type oxidase (T4_02432–5) were significantly upregulated 6–10-fold and ~2.5-fold, respectively, in the anoxic CH_3OH + N_2O-grown cells in comparison to the O_2-replete CH_3OH + O_2-grown cells (Fig. 6, Supplementary Dataset 6). Interestingly, the anoxic CH_3OH + N_2O-grown cells also significantly upregulated the expression of genes encoding one of its low-affinity *aa*$_3$-type oxidases (T4_00359–61) by 17- to 60-fold in comparison to the O_2-replete CH_3OH + O_2-grown cells (Fig. 6, Supplementary Datasets 5, 6) as well. In the case of strain IT6 cells, transcriptional levels for genes encoding the high-affinity *cbb*$_3$ TO were not significantly different between cells grown under O_2-replete CH_3OH + O_2 and anoxic CH_3OH + N_2O conditions (Supplementary Fig. 10, Supplementary Dataset 7). There was a 6- to 30-fold upregulation of the expression of the genes encoding the three subunits of *ba*$_3$ medium-affinity TO (IT6_00283–5) in anoxic CH_3OH + N_2O-grown cells of strain IT6 (Supplementary Fig. 10, Supplementary Dataset 7). Also upregulated in the anoxic CH_3OH + N_2O-grown cells of strain IT6 (2.8–172-fold) were genes for low-affinity *aa*$_3$-type TO in the cluster IT6_00893–9, subunit I of *aa*$_3$-type TO (*ctaD*; IT6_01499), and an accessory protein of *aa*$_3$-type TO (*ctaA*; IT6_02050) (Supplementary Fig. 10, Supplementary Dataset 7).

Further, the transcript levels of the TO-encoding genes were compared in strain T4 cells grown under suboxic CH_4 + O_2 + N_2O and O_2-replete CH_4 + O_2 conditions (Supplementary Dataset 5). The genes in the operon T4_02429–35 encode a high-affinity *cbb*$_3$-type oxidase that was upregulated 4 to

40-fold in suboxic CH_4 + O_2 + N_2O-grown cells compared to the O_2-replete CH_4 + O_2-grown cells (Supplementary Fig. 9, Supplementary Datasets 5, 6). In addition, the suboxic CH_4 + O_2 + N_2O-grown cells increased the expression of some genes encoding a low-affinity *aa*$_3$-type oxidase (T4_00956–8) by 2.6 to 5-fold (Supplementary Fig. 9, Supplementary Datasets 5, 6).

The upregulation in T4 strain cells of genes encoding high-affinity *cbb*$_3$-type and *bd*-type TOs in suboxic and anoxic conditions, respectively, is consistent with studies indicating that these oxidases are used by microorganisms to conserve energy in low-oxygen environments[12, 15, 16]. On the other hand, the observed expression and upregulation of genes encoding low-affinity TOs in strain T4 at suboxic conditions is unexpected. However, some evidence suggests this is not uncommon: analysis of the transcriptome of *Methyloprofundus* sp. INp10, a methanotrophic endosymbiont of deep-sea bathymodiolin mussels, revealed the *aa*$_3$ low affinity-TO as the most used TO in hypoxia[17]. Some *Acidobacteria* strains were found to grow in suboxic conditions with low-affinity TOs at O_2 concentrations as low as 1 nM (ref.[18]). Likewise, the low-affinity *aa*$_3$-type TO of the ammonia-oxidizing bacterium, *Nitrosomonas europaea*, was significantly upregulated during suboxic growth conditions[19]. Oxygen deprivation led to an increase in the expression of genes encoding low-affinity TOs in both the T4 and IT6 strains. Because low-affinity TOs are more efficient at generating ATP than high-affinity TOs[20-22], their utilization may be ecologically beneficial to microorganisms in low-oxygen ecosystems. Furthermore, the upregulation of low-affinity TOs coincided with the upregulation of denitrification genes, including the N_2OR genes, which also coincided with the upregulation of methane monooxygenase. Thus, especially in methanotrophs, the upregulation of low-affinity TOs in suboxic conditions may help the redistribution of the limited O_2 to the methane monooxygenase, especially in the presence of other terminal electron acceptors, such as N_2O.

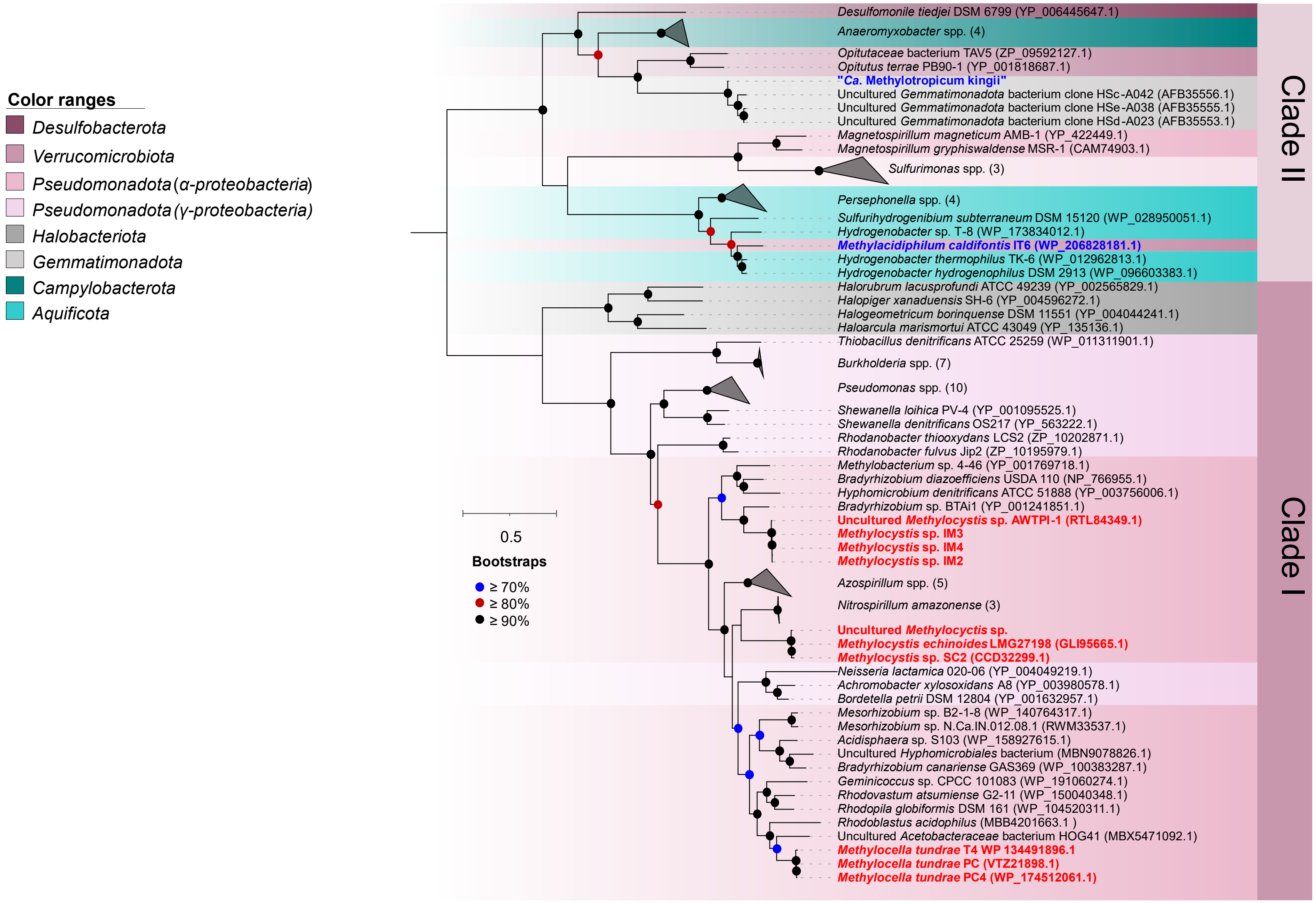

Color ranges
Desulfobacterota
Verrucomicrobiota
Pseudomonadota (α-proteobacteria)
Pseudomonadota (γ-proteobacteria)
Halobacteriota
Gemmatimonadota
Campylobacterota
Aquificota
0.5
Bootstraps
≥ 70%
≥ 80%
≥ 90%
Desulfomonile tiedjei DSM 6799 (YP_006445647.1)
Anaeromyxobacter spp. (4)
Opitutaceae bacterium TAV5 (ZP_09592127.1)
Opitutus terrae PB90-1 (YP_001818687.1)
"Ca. Methylotropicum kingii"
Uncultured Gemmatimonadota bacterium clone HSc-A042 (AFB35556.1)
Uncultured Gemmatimonadota bacterium clone HSe-A038 (AFB35555.1)
Uncultured Gemmatimonadota bacterium clone HSd-A023 (AFB35553.1)
Magnetospirillum magneticum AMB-1 (YP_422449.1)
Magnetospirillum gryphiswaldense MSR-1 (CAM74903.1)
Sulfurimonas spp. (3)
Persephonella spp. (4)
Sulfurihydrogenibium subterraneum DSM 15120 (WP_028950051.1)
Hydrogenobacter sp. T-8 (WP_173834012.1)
Methylacidiphilum caldifontis IT6 (WP_206828181.1)
Hydrogenobacter thermophilus TK-6 (WP_012962813.1)
Hydrogenobacter hydrogenophilus DSM 2913 (WP_096603383.1)
Halorubrum lacusprofundi ATCC 49239 (YP_002565829.1)
Halopiger xanaduensis SH-6 (YP_004596272.1)
Halogeometricum borinquense DSM 11551 (YP_004044241.1)
Haloarcula marismortui ATCC 43049 (YP_135136.1)
Thiobacillus denitrificans ATCC 25259 (WP_011311901.1)
Burkholderia spp. (7)
Pseudomonas spp. (10)
Shewanella loihica PV-4 (YP_001095525.1)
Shewanella denitrificans OS217 (YP_563222.1)
Rhodanobacter thiooxydans LCS2 (ZP_10202871.1)
Rhodanobacter fulvus Jip2 (ZP_10195979.1)
Methylobacterium sp. 4-46 (YP_001769718.1)
Bradyrhizobium diazoefficiens USDA 110 (NP_766955.1)
Hyphomicrobium denitrificans ATCC 51888 (YP_003756006.1)
Bradyrhizobium sp. BTAi1 (YP_001241851.1)
Uncultured Methylocystis sp. AWTPI-1 (RTL84349.1)
Methylocystis sp. IM3
Methylocystis sp. IM4
Methylocystis sp. IM2
Azospirillum spp. (5)
Nitrospirillum amazonense (3)
Uncultured Methylocyctis sp.
Methylocystis echinoides LMG27198 (GLI95665.1)
Methylocystis sp. SC2 (CCD32299.1)
Neisseria lactamica 020-06 (YP_004049219.1)
Achromobacter xylosoxidans A8 (YP_003980578.1)
Bordetella petrii DSM 12804 (YP_001632957.1)
Mesorhizobium sp. B2-1-8 (WP_140764317.1)
Mesorhizobium sp. N.Ca.IN.012.08.1 (RWM33537.1)
Acidisphaera sp. S103 (WP_158927615.1)
Uncultured Hyphomicrobiales bacterium (MBN9078826.1)
Bradyrhizobium canariense GAS369 (WP_100383287.1)
Geminicoccus sp. CPCC 101083 (WP_191060274.1)
Rhodovastum atsumiense G2-11 (WP_150040348.1)
Rhodopila globiformis DSM 161 (WP_104520311.1)
Rhodoblastus acidophilus (MBB4201663.1)
Uncultured Acetobacteraceae bacterium HOG41 (MBX5471092.1)
Methylocella tundrae T4 WP 134491896.1
Methylocella tundrae PC (VTZ21898.1)
Methylocella tundrae PC4 (WP_174512061.1)
Clade II
Clade I

Supplementary Figure 1. Phylogenetic reconstruction of NosZ proteins encoded in methanotrophs and other prokaryotes. A maximum-likelihood tree was inferred with IQ-TREE using ModelFinderPlus (IQ-TREE options: -m MFP, -B 1000) and rooted at the mid-point. Methanotrophs with clade I and clade II NosZ proteins have their names written in red and blue text, respectively. Bootstrap values ≥ 70% based on 1,000 replications are indicated. The scale bar represents a 0.5 change in each amino acid position.

6

```
Halorubrum lacusprofundi ATCC 49239 (YP_002565829.1)          YTWGDTHHPRVSQTDNDYDGRWLFVNDKANGRMARVDLEYFETDAIT-NVPNCQGVHGACMQL--------PDTQLVFGVGEFRVPMPND-GRDV---QSPDEYGSVLSAINPETMDV----EWQVE---VDGNMDNGDGGKEGRWFFATGYNSEEGV---------TESEMARSDRDYV    151
Thiobacillus denitrificans ATCC 25259 (YP_315147.1)          YTTGDTHHVHQSYRDGTYDGKYMFVNDKIHGRLARIRMDTMECDKII-ELPNVQGFHGTFTDKRDPVDASINHTTRVFCGGEFGIPLPND-GRDL---EDVSKYKSVFSCVDAESMEM----RWQVL---IDGNCDLVASSYDGKLAATNQYNTENGA---------RYEDMMSAEMDAC    159
Burkholderia mallei ATCC 23344 (YP_102711.1)                 YFTGDTHHVHMSYTDGTYDGKYIFVNDKINARIARVRLDTMECDAIT-PLPNVQGFHGIFPDKRDPLDQTINYTTRVFCGNEFHIPHPND-GRDL---NDPKKYYCLFTCVDAKTMQP----RWQVK---IDGNMDLVATSYDGKLACSNQYNTENGV---------HYEEMMAAERDAC    159
Bradyrhizobium sp. BTAi1 (YP_001241851.1)                     WMNGDCHHPHMSFTNGTYDGRYVFINDKANTRLARIRTDVMKCDKII-ELPNQYTIHGMRPQK-------FPRTGYLFCNGEYEVPLPND-GKVL---DDKSKYWSIFSAVDGDTMKV----AWQVI---VDGNLDNTDCDYQGKYAFATCYNSEKGV---------TLAEMTASEQDWV    152
Hyphomicrobium denitrificans ATCC 51888 (YP_003756006.1)     YMNGDLHHPHISFTDGTYDGRYAFMNDKANTRVARVRLDVMKCDKII-QLPNQHTVHGLRVQK-------YPRTGYVFCNGEDGVPLPND-GKVL---NSPKDYYSIFTAVDGDTMKV----AWQVI---VDGNLDNVDADYQGKYAFSTCYNSEGGV---------TLAEMTAKEQDWL    152
Neisseria lactamica 020-06 (YP_004049219.1)                   YPNGDLHHPHLSFTDQTYDGRYAYANDKANNRVCRVRLDVMKADKII-DIPNASGIHGLRPQR-------YPKTGYVFANGEHITPVSGV-GKL----DDAKTWNAVYTAIDGETMEI----AWQVL---VDGNLDNGDADYQGKYSFATCYNSERAL---------TVQGASSNEQDWC    151
Bordetella petrii DSM 12804 (YP_001632957.1)                  YLNGDAHHPHMSFTDRTYDGRYIFINDKANDRVARIRCDVMKTDKII-EIPNASGIHGLRPQR-------YPRTGYVFANGEHIVPLPND-GTVT--DDPKKNFFAIYSAIDGDTMKV----AWQVM---VDGNLDNGDADYQGKYSFATCYNSEKGL---------SVEEASANEQDWV    153
Achromobacter_xylosoxidans A8 (YP_003980578.1)                FINGDAHHPHLSFTDRTYDGRYLYINDKANNRVARIRLDVMKTDKIT-ELPNVSGVHGLRPQR-------YPRTGYVFANGEHIIPLPND-GKVT--DDPKKNFFAIYTALDGDTMKV----AWQVL---VDGNLDNGDADYQGKYSFSTCYNSEKGV---------TVEEASANEQDWV    153
Rhodanobacter fulvus Jip2 (ZP_10195979.1)                     -PGGDCHHPHMSQTDGHYDGRYIFINDKAHTRVARIRCDVMRTDRII-DVPNVQAIHGLRVQR-------APRTGYVFANGEFMIPAPND-GRD---LEDPKKYQTMFSAIDGDTMEV----AWQVL---VDGNLDNTEADYAGKYAISTCYNSEGGL---------DLPGTMQAERDWA    151
Shewanella loihica PV-4 (YP_001095525.1)                      LMAGDAHHPHMSMQDGRYDGKYLFINDKANTRVARIRCDVMKTDKMI-TIPNAQAIHGLRVQK-------VPYTKYVICNGEFEVPLNNN-GKET--LEDVSTYRSLFNVIDAEKMEV----AFQVM---VDGNLDNTDADYDGKYFASTCYNSEMGM---------NLSEMITAERDHV    153
Pseudomonas aeruginosa UCBPP-PA14 (YP_789757.1)               FLNGDCHHPHISMTDGKYDGKYLFINDKANSRVARIRLDVMKCDRIV-TIPNVQAIHGLRLQK-------VPHTRYVFCNAEFIIPHPND-GSTF--DLSGDNAFTLYNAIDAETMEV----AWQVI---VDGNLDNTDMDYSGRFAASTCYNSEKAV---------DLGGMMRNERDWV    153
Methylobacterium sp. 4-46 (YP_001769718.1)                    YHNGDLHHPHLSFTDGTYDGRYCFVNDKANTRVARIRLDVMRCDKII-ELPNQHTVHGLRVQK-------YPRTGYVFCNGEDRVPLPND-GKVL---DDNKQYHAIFTAVDGDTMQV----AWQVL---VDGNLDNVDADYQGKYAFATCYNSEEGV---------TLAEMTANEQDWV    152
Methylocystis sp. IM2/IM3/IM4                                 WMNGDAHHPHMSFTDGTYDGRYIFINDKANTRLARIRTDIMKCDKII-ELPNQYTVHGMRPQK-------FPRTGYLFCNGEYEVPIPND-GKTL---DDPKQYRSIFSAVDGDSMKV----AWQVI---VDGNLDNNDADYQGKYAFATCYNSEKGV---------NLAEMTAKEQDWV    152
Methylocystis sp. AWTPI-1 (RTL84349.1)                        WMNGDAHHPHMSFTDGTYDGRYIFINDKANTRLARIRTDIMKCDKII-ELPNQYTVHGIRPQK-------FPRTGYLFCNGEYEVPIPND-GKTL---DDPKQYRSIFSAVDGDSMKV----AWQVI---VDGNLDNNDADYQGKYAFATCYNSEKGV---------NLAEMTAKEQDWV    152
Methylocystis echinoides LMG27198 (GLI95665.1)                YQNGDLHHPHMSFTDGTYDGRYIFINDKANCRVARIRCDIMKTDKII-EIPNVTDIHGLRLQK-------FPRTGYVFANGEHIVPLPND-GKNHAATEDKKKYADFFTAIDGDTMKI----AWQVQ---IDGNLDNCDADYTGAYAFSTCYNSEMGF---------TSADMTSSPQDWA    155
Methylocystis sp. NODE95                                       YQNGDLHHPHMSFTDGTYDGRYIFINDKANCRVARIRCDIMKTDKII-EIPNVTDIHGLRLQK-------FPRTGYVFANGEHIVPVPND-GKNHAATEDKKKYADFFTAIDGDTMKI----AWQVQ---IDGNLDNCDADYTGAYAFSTCYNSEMGF---------TSADMTSSPQDWA    155
Methylocystis sp. SC2 (CCD32299.1)                            YQNGDLHHPHMSFTDGTYDGRYIFINDKANCRVARIRCDIMKTDKII-EIPNVTDIHGLRLQK-------FPRTGYVFANGEHIVPLAND-GKNHAAMEDKKKYADFFTAIDGDTMKI----AWQVQ---IDGNLDNCDADYTGAYAFSTCYNSEMGF---------TSADMTSSPQDWA    155
Methylocella tundrae PC4(WP_174512061_1)                       WQNGDLHHPHMSFTDGTYDGRFIFVNDKANCRIARIRCDVMKTDKII-EIPNASDIHGLRPQK-------YPRTGYVFANSEHVIPIPND-GHVL--DDPKNNYWSVYTAVDGDTMKV----AWQVL---VDGNLDNTDCDYQGKYAFSTCYNPNKGV---------TLAEMTDHEQAWA    153
Methylocella_tundrae T4 (WP_134491896.1)                       WQNGDLHHPHMSFTDGTYDGRFIFVNDKANCRIARIRCDVMKTDKII-EIPNASDIHGLRPQK-------YPRTGYVFANSEHVIPIPND-GHVL--DDPKNNYWSVYTAVDGDTMKV----AWQVL---VDGNLDNTDCDYQGKYAFSTCYNPNKGT---------TLAEMTDHEQAWV    153
Sulfurimonas_autotrophica DSM 16294 (YP_003893043.1)          INWGDTHHPAISETNGVYDGKWLAINDKANPRIAILDLNDFETKQIV-VNPVFKSDHGGAFFT--------PNSEYILEASQYAAPFDNN-YHPIEEYKETYRGGITFWKFNDKIGRINQKKSFVLELPPYMQDLSDAGKGASNGWGFTNSFNSEMYTGGIEVGMPPFEAGCSRNDTDFL    170
Sulfurimonas_denitrificans DSM_1251 (YP_393811.1)             INWGDTHHPALSEKDGKYDGKWLAINDKANPRIAIIDLADFETKQIV-VNPVFKSAHGGAFFT--------QNSDYIIEACQYAAPLDNN-YHPIEDYKEAYRGGATMWRFDPAKGKINVKESFTIEMPPYMQDLSDSGKGVSDGWGFTNSFNSEMYTGGIEVGMPPNEAGMSRNDTDFL    170
Persephonella_marina (WP_012675862.1)                         IPWGDTHHPELSQTNGVPDGRWIFINENNTPRVARIDLRTFETIEIL-EIPNSGGNHASAFVT--------PNTEYITAGTRFSVPIPQK-DVPIASYAKNFKGTLTFIRAD-KPGKMDI--AFQILVPGYDYDLAHCGKGPSYGWCFYTSYNTEQAH-------TLLEINASQKDKDYI    160
Hydrogenobacter sp. T-8 (WP_173834012.1)                      IPWDDTHHPWLSQTNGEHDGRWLFINANNTPRIARIDLTTFETTEII-EIPNSAGNHPSPYGT--------ENTEYVVAGTRFSVPVPQK-DVPIDSYAQNFKGTISFIKAN-EPGKMDV--AFQILMPGFNYDLARCGRGPSHGWCFFTSYNTEQAH-------TLLEINASKNDKDYI    160
Methylacidiphilum caldifontis IT6 (WP_206828181.1)            IPWDDSHHTELSQTDGVPNGRWLFINANNTPRIARIDLFSFVTKEIL-EIPNTAGNHASPFIT--------PDSKYITAATRFSIPIPQK-DVPISSYAENFKGTLSFIKAD-EPGKMDV--AFQILMPGFDYDLAHCGKGPSYGWAFFTCYNTEQAY-------TLLEINASRNDKDYI    160
Hydrogenobacter_hydrogenophilus_(WP_096603383.1)              IPWDDSHHSELSQTDGVPDGRWLFINANNVPRIARIDLTTFETTEII-EIPNSAGNHASPFIT--------PDSKYLTAATRFSVPIPQK-DVPISSYAENFKGTISFVKAD-EPGKMDV--AFQILVPGYDYDLAHCGKGPSYGWCFFTSYNTEQAY-------TLLEINASKNDKDYI    160
Hydrogenobacter thermophilus TK-6 (WP_012962813.1)            VPWDDSHHPELSQTDGVPDGRWLFINANNVPRIARIDLSTFETTEII-EIPNSAGNHASPFIT--------PDSKYVTAATRFSVPIPQK-DVPISSYAENFKGTLSFIKAN-EPGKMDV--AFQILVPGYDYDLAHCGKGPSYGWCFFTSYNTEQAY-------TLLEINASKNDKDYI    160
Opitutus terrae PB90-1 (YP_001818687.1)                        YTWGDVHHPGLSQTDGKYDGRWLFVNDNANNRIARVDLRDFKTKQILGPIPNSSGNHGSSFVT--------ENTENVLVATRFSVPLPKGRYAELNDYEKEFNGMVSGIKVDPQSGEMSV--GWQILTPPFDWDLGSTGKGPSSGWAFWTSYNTEMAH-------DQLEVNASQRERDYA    163
Desulfomonile tiedjei DSM 6799 (YP_006445647.1)                LTWGDTHHPGLSETKGEYDGRWLFINDNAHGRVARIDLRDFKTKQIFGPIPNLAGNHASVYLT--------WNTEYLFGATRFSIPIPKGTAADVKDYKEKYKGVISAIAVDPKDGTMSL--GWQIVMPPWNYDVADAGKLVSGDWWFVTTYNTEAEF----IDGKPLEITASQRDKDYI    166
Anaeromyxobacter dehalogenans 2CP-1 (YP_002491966.1)           LTWGDVHHPALSETGGDYDGRWLFVNE-MNGRVARIDLRDFKTRQIIGPVPNISGNHGSTFIT--------PNSEYILMSSRFSIPIPKGKAVSIDRYASEYKGVAAGIKVDPKTGQMSL--GWQVLLPPFDWDLGDAGKKLSEGWFFLTCYNSERAT-------GKLEVTASQRDRDYI    162
Anaeromyxobacter sp. Fw109-5 (YP_001377446.1)                  LTWGDVHHPALSETKGDYDGRWLFVNE-MNGRIARIDLRDFKTRQIIGPVPNVSGNHGSAFVT--------PNTEYAMMSSRFSIPIPKGTAVSIDKYASEYKGIVAGIKIDPESGKMSL--GWQVALPPFDWDLGDAGKLVSDGWMFLTCYNSERGT-------GKLEVTASQRDRDYI    162
                                                                  .* **   *     :*::   *     *:. :     :    :     *     *                    :        .   *                             :               : :        :                . :* :

Halorubrum lacusprofundi ATCC 49239 (YP_002565829.1)          KAFDIPAIWDAVEAGNYEEIS---------------------G--VPVVDGTMDSSLNEGDRPIVRYVATPKSPHGISVTPDGNYAIASGKLDPSCTVIDIDAIAEVDD----------------PQESIVGQPRIGMGPLHTAYDG---RGHAYTTLFIDSQVAKWDYEAAVEAEAGS    288
Thiobacillus denitrificans ATCC 25259 (YP_315147.1)          LFFNIARIEEAVKAGKVTYIA---------------------GSKVPVVDGTRAANA-DPKTALTCYVPVSKNPHGVNISPDGKYYACSGKLSPTATVIEHALVLKWFDGALA------------KPRDAVVAEAEIGLGPLHTGFDN---KGNAYTTLFLDSQIVKWNVEAAIKSFKGD    302
Burkholderia mallei ATCC 23344 (YP_102711.1)                 VFFNVARIEEAVKNGKFFKVG---------------------NSPVPVVDGTRAANA-DPKTALTCYVSVPKNPHGVNASPDGKYYACSGKLSPTATLISHELMLKWFAGELK------------EPRDAVVAEPEIGLGPLHTGFDG---KGYAYTTLFLDSQIVKWSLEEAIKAYKGE    302
Bradyrhizobium sp. BTAi1 (YP_001241851.1)                     TIFNIKRIEDAVKKGDIKEMN---------------------G--VPVIDGRKG-------SPYTRYVPVSNSPHGLNTAPDGIHVVANGKLAPTVTVMDVRLIDSLFDDKIK-------------PRDIIVAEPELGLGPLHTAFDG---RGNAYTTLFLDSQVCKWSLDLAKRAFKGE    286
Hyphomicrobium denitrificans ATCC 51888 (YP_003756006.1)     TVFNIKRIEEAVKKGDFKEMG---------------------G--VPVIDGRKG-------SPYTRYIPVSNGPHGINTAPDGIHIIANGKLSPTCTVFDVRKFDDLFDDKIK-------------PRDTVVAEPELGLGPLHTAYDG---KGNCYTTLFIDSQICKWNLDLAKRAYAGE    286
Neisseria lactamica 020-06 (YP_004049219.1)                   VVFDLKAIEEGIKAGDFKEVN---------------------G--VKMLDGRAE-----AKSKYTRYIPVPNSPHGCNASPDGKYIMLNGKLSPTVTVLDVSKLDDLFAGKIK-------------ERDVVVAEPQLGLGPLHTAFDG---RGNAYTTLFIDSQMVKWNIDDAIKAYKGD    287
Bordetella petrii DSM 12804 (YP_001632957.1)                  VVFNIKRIEEAVKSGDFKMMN---------------------G--VPVVDGRHG-------SKYTRYIPVPNSPHGCNAAPDGIHVVLNGKLSPTVTVLDVRKLDDLFDGKIK-------------ERDCVVAEPQLGLGPLHTAFDG---RGNAYTTLFIDSQMVKWNIDKARRAYLGE    287
Achromobacter_xylosoxidans A8 (YP_003980578.1)                VVFNIKRIEEAVKKGDFKTMG---------------------G--VPVIDGRHG-------SKYTRYIPVPNSPHGCNAAPDGIHLVLNGKLSPTVTVLDVRTLDDLFDDKIK-------------PRDCVVAEPQLGLGPLHTAFDG---RGNAYTTLFIDSQMVKWNIDKAKRAFKGE    287
Rhodanobacter fulvus Jip2 (ZP_10195979.1)                     VVFNIARIEEAVKQGKFKTIG---------------------DSKVPVLDGRHG-------SALTLYIPIPKNPHGINASPDGKYVVANGKLSPTVSVIEWSRIDDCFAGKLK------------DPRDTVVAEPELGLGPLHTAYDG---RGNAYTTLFIDSQIAKWNIADAIAAHNGK    288
Shewanella loihica PV-4 (YP_001095525.1)                      VIFNLARCEAAVKAGKFKTYN---------------------GNKIPVLDGRKG-------SDLTRYIPVPKSPHGLNTSPSGKYFVANGKLSPTVSIISIEKLDDLFDDKIK-------------PRDAIVGEPELGLGPLHTAFDN---KGYAYTTLFIDSQIAKWNVEEAIQAYQGK    289
Pseudomonas aeruginosa UCBPP-PA14 (YP_789757.1)               VVFDIPRIEAEIKAKRFVTLG---------------------DSKVPVVDGRRKDG---KDSPVTRYIPVPKNPHGLNTSPDGKYFIANGKLSPTCTMIAIERLGDLFAGKLA------------DPRDVVVGEPELGLGPLHTTFDG---RGNAYTTLFIDSQLVKWNLADAVRAYKGE    294
Methylobacterium sp. 4-46 (YP_001769718.1)                    VIFNIKRIEEAVRKGEAKVVG---------------------G--VPMVDGRHG-------SRFTRYVPVSNSPHGMNTAPDGIHVVANGKLSPTVTVMDVRLFDDLFDDKIK-------------PRDVVVAEPELGLGPLHTAYDG---KGNAFTTLFIDSQVVKWNIEKARQAYKGA    286
Methylocystis sp. IM2/IM3/IM4                                 VIFSIKRIEDAVKKGHFSRIN---------------------G--VPVIDGRHG-------SPYTRYVPVSNNPHGMNAAPDGIHVVANGKLSPTVTVFDVRLFDQLFDDKLK-------------PRDVVVAEPELGLGPLHTAFDG---RGNAYTTLFLDSQVCKWSIDKAIKAFKGE    286
Methylocystis sp. AWTPI-1 (RTL84349.1)                        VIFSIKRIEDAVKKGHFSRIN---------------------G--VPVIDGRHG-------SPYTRYVPVSNNPHGMNAAPDGIHVVANGKLSPTVTVFDVRLFDQLFDDKLK-------------PRDVVVAEPELGLGPLHTAFDG---RGNAYTTLFLDSQVCKWSIDKAVKAFKGE    286
Methylocystis echinoides LMG27198 (GLI95665.1)                VIFNIKRIEEAIKKGDYKEIG---------------------G--VRVVDGRHG-------SAYTRYVPISNNPHGCNMAPDGKHLAINGKLSPTVSLLDVTLFDDLFDDKIK-------------PRDVVVAEPELGLGPLHTAFDG---RGNAFTTVFIDSQIVKWNIDKARRAFKGE    289
Methylocystis sp. NODE95                                       VIFNIKRIEEAIKKGDYKEIG---------------------G--VRVVDGRHG-------SPYTRYVPISNNPHGCNMAPDGKHLAINGKLSPTVSLLDVTLFDDLFDDKIK-------------PRDVVVAEPELGLGPLHTAFDG---RGNAYTTVFIDSQIVKWNIDKARRAFKGE    289
Methylocystis sp. SC2 (CCD32299.1)                            VIFNIKRIEEAIKKGDYKEIG---------------------G--VRVVDGRHG-------SPYTRYVPISNNPHGCNMAPDGKHLAINGKLSPTVSLLDVTLFDDLFDDKIK-------------PRDVVVAEPELGLGPLHTAFDG---RGNAYTTVFIDSQIVKWNIDKARGAFKGE    289
Methylocella tundrae PC4(WP_174512061_1)                       TIFNIKRIEEAVKNGDYKEMN---------------------G--VPVVDGRHG-------SKYTRYIPIPNSPHGVNTAPDGIHVVINGKLSPTVSVMDVRKLDDLFDDKIK-------------PRDVIVAEPEVGLGPLHTAFDG---RGNAFTTTFIDSQMVMWNIDKAKRAFAGE    287
Methylocella_tundrae T4 (WP_134491896.1)                       TIFNIKRIEEAVKSGDYKEMS---------------------G--VPVVDGRHG-------SKYTRYIPIPNSPHGVNTAPDGIHVVINGKLSPTVSVMDVRKLDDLFDDKIK-------------PRDVIVAEPEVGLGPLHTAFDG---RGNAFTTTFIDSQMVMWNIDKAKRAFAGE    287
Sulfurimonas_autotrophica DSM 16294 (YP_003893043.1)          HVYNWKKLATLTKNKKNYKVIN--------------------GIKVIPMSVAVA-------NNALFLIPEPKSPHGVDVSPDGEYIVVCGKLDTHASVYKWSKIQKLIKDHKYAGKDPYGIPILSMKDSLHGQVEL-GLGPLHNQYGKHWKDGEIYTSLYVDSQIVKWDYK---------    313
Sulfurimonas_denitrificans DSM_1251 (YP_393811.1)             HVYNWKKLAELAKDSKNVKIIN--------------------DHKVIPMDIAVK-------NNALFLIPEPKSPHGVDVSPDGEYITVCGKLDTHASVYKWSNIKKLIDAKEYAGKDPYGIPILDMKKSLHGQAEL-GLGPLHNQYSN--IDGEIYTSLYVDSQIVKWNYK---------    311
Persephonella_marina (WP_012675862.1)                         AAVNWKRAEQCIAEGKYHVMKTEYYHNYIPDPEGDRIAVSEVKKEVKVIIPKEC-------KGVLYFLPTPKSPHGVDVDPTGEYIIAGGKLSTVLPIHSFSKMIKAIKNKDFVGEV-DGIPILRYEAISYCEVPNPCLGPLHTEFDG---KGYSYTSCFITSEVIKVDYK---------    320
Hydrogenobacter sp. T-8 (WP_173834012.1)                      AAINWKRAEECLSQGRFQEVPAVYYHNYYD--EKKHMAVSEVKKSVKMINPKDC-------PGVAYLLPTPKSPHGVDVSDDGEYIVGGGKLATVIPIHSFSKMLKAIEQKAFEKEI-DGVPVLKYDAVLHCEVEKPCLGPLHTEFDG---KGFAYTSCFVSSEVVKYDYK---------    318
Methylacidiphilum caldifontis IT6 (WP_206828181.1)            AAVNWKKAEEYIAQGKYQEMPTVYYHNYWD--EKKHMAFSEVKKSVKVINPKEC-------PGVVYYLPTPKSPHGVDVSPDGEYIVGNGKLATVIPIHSFSKMLKAIEQKAFEKEV-DGIPVLKYDAVLHCEIQKPGLGPLHTEFDG---KGFAYTSCFVSSEVVKYDYK---------    318
Hydrogenobacter_hydrogenophilus_(WP_096603383.1)              AAVNWKRAEECIAQGKYQEMPATYYHNFYD--EKKHMAVSEVKKSVKVIIPKDC-------PGVIYYLPTPKSPHGVDVSPDGEYIIGGGKLATVIPIHSFSKMLKAIEQKAFEKEV-DGIPVLKYDAVLHCEVPKPCLGPLHTEFDG---KGFAYTSCFVSSEVVKYDYK---------    318
Hydrogenobacter thermophilus TK-6 (WP_012962813.1)            AAVNWKRAEECIAQGKYQEIPATYYHNYYD--EKKHMAFSEVKKSVKVIIPKDC-------PGVMYYLPTPKSPHGVDVSPDGEYIIGGGKLATVIPIHSFSKMLKAIEQKAFEKEV-DGIPVLKYDAVLHCEVPKPCLGPLHTEFDG---KGFAYTSCFVSSEVVKYDYK---------    318
Opitutus terrae PB90-1 (YP_001818687.1)                        AVVNWKAAEAAVREGKGTDMD-------------------------GVPVIDPAKV-------PGVLYFLPVPKSPHGIDVDPSGRWIAAGGKLQPTASVFDFEKIRVAIEKRDFEGEF-RGVPILRFESVLEGEVPV-GLGPLHTQYDG---KGNAYTSLFIESVVTKWKLPPWTDEQKKD    308
Desulfomonile tiedjei DSM 6799 (YP_006445647.1)                IAVNWKGVEGEIKKGNFTEID-------------------------GAKVFDPVKT-------PGFVFAVPCAKSPHGVDVSPDGKWFIGNGKLSPTVSIFSFEKLQKAIQEKNFAQHD-MGIPVVKFEAVTEADIPV-GLGPLHTVFDD---KGFAYTSLFVESAVAKWQWGGG------G    305
Anaeromyxobacter dehalogenans 2CP-1 (YP_002491966.1)           AAIDWRLAEKAAAEGKGELVG-------------------------GVKVLDPKTV-------PGLVYLLPCGKSPHGVDVSPDGKYVVGSGKLQGVTTAFNFEKVLTAIKNKDFAGEE-DGIPVLKYESIKDAEVPV-GLGPLHTQFGP---DGMAYTSLFVDSALAKWKLG---------    298
Anaeromyxobacter sp. Fw109-5 (YP_001377446.1)                  AAINWREAEKAAAEGKGDLIG-------------------------GVKVLDPRKV-------PGLVYLMPCGKSPHGVDVSPDGKYIIGSGKLQGVTTAFNFEKVLTAIKNKDFTGEE-DGIPVLKYESIKDAEVPV-GLGPLHTQFGP---DGWAYTSLFVDSALAKWKLG---------    298
                                                                 .                                                          .                   :   :.*** .     *       ***         .                            :****. :.      *  :*: :: * :    .
```

7

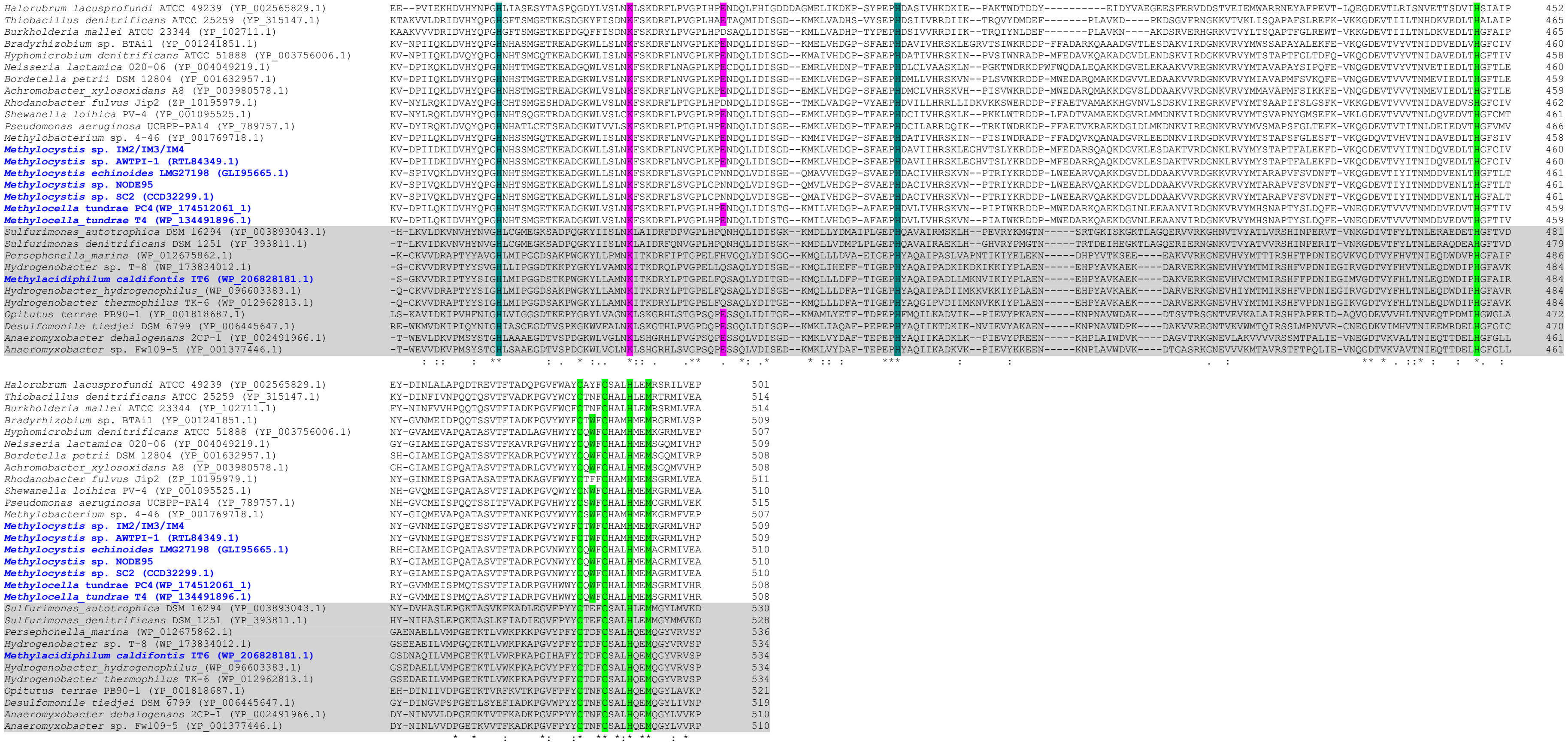

Supplementary Figure 2. Sequence conservation across NosZ homologues. Conserved histidine residues of the beta-propeller domain implicated in Cu$_Z$-center coordination are highlighted in teal green. Residues involved in binding the Cu$_A$-center of the C-terminal cupredoxin domain are highlighted in bright green. Calcium binding residues, involved in NosZ dimerization, are highlighted in pink. Amino acid conservation between the proteins is indicated below the alignments with the following symbols in parentheses: (*****), 100% identical amino acid residues; (**:**), conserved substitutions; (**.**), semi-conserved substitutions. NosZ proteins highlighted in grey are clade II type, while unhighlighted are clade I type. Names of NosZ-containing methanotrophs are written in blue text.

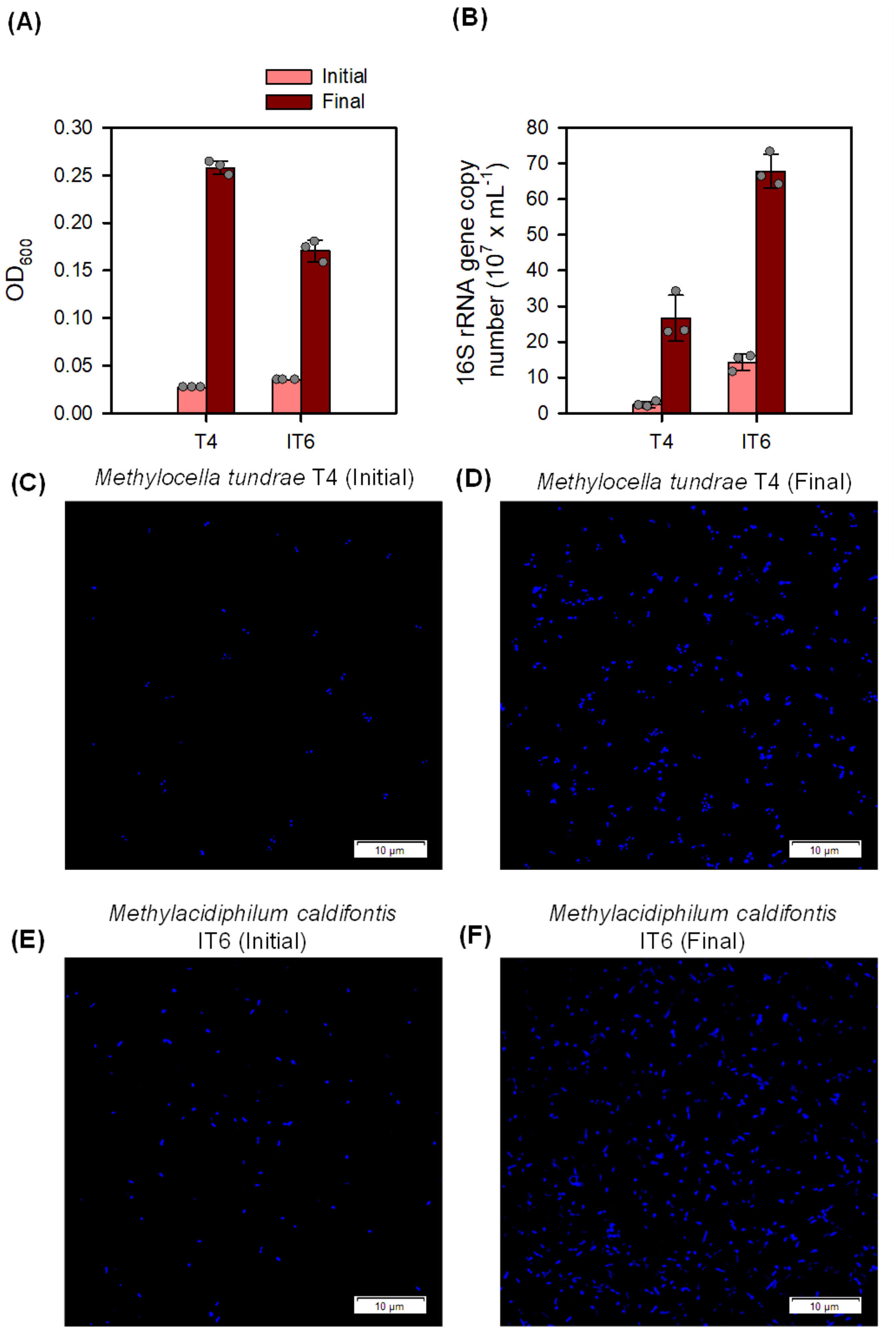

Supplementary Figure 3. Anaerobic growth of *Methylocella tundrae* T4 and *Methylacidiphilum caldifontis* IT6 with methanol as the electron donor and N_2O as the terminal electron acceptor.

Anaerobic cell growth was monitored using (**A**) optical density (OD_{600}) measurements, (**B**) qPCR to quantify bacterial 16S rRNA gene copy numbers, and (**C-F**) fluorescence microscopy to count DAPI-stained cells. The cells used for the measurements were obtained on day 0 (initial) and approximately on day 7 and day 15 (final) for *Methylocella tundrae* T4 and *Methylacidiphilum caldifontis* IT6, respectively. All experiments were performed in triplicates. Data are presented as mean ± 1 standard deviation (SD), and the error bars are hidden when they are smaller than the width of the symbols. The fluorescence microscopy experiments were performed on triplicate filters with similar results. Scale bar, 10 μm. Source data are provided as Source Data file.

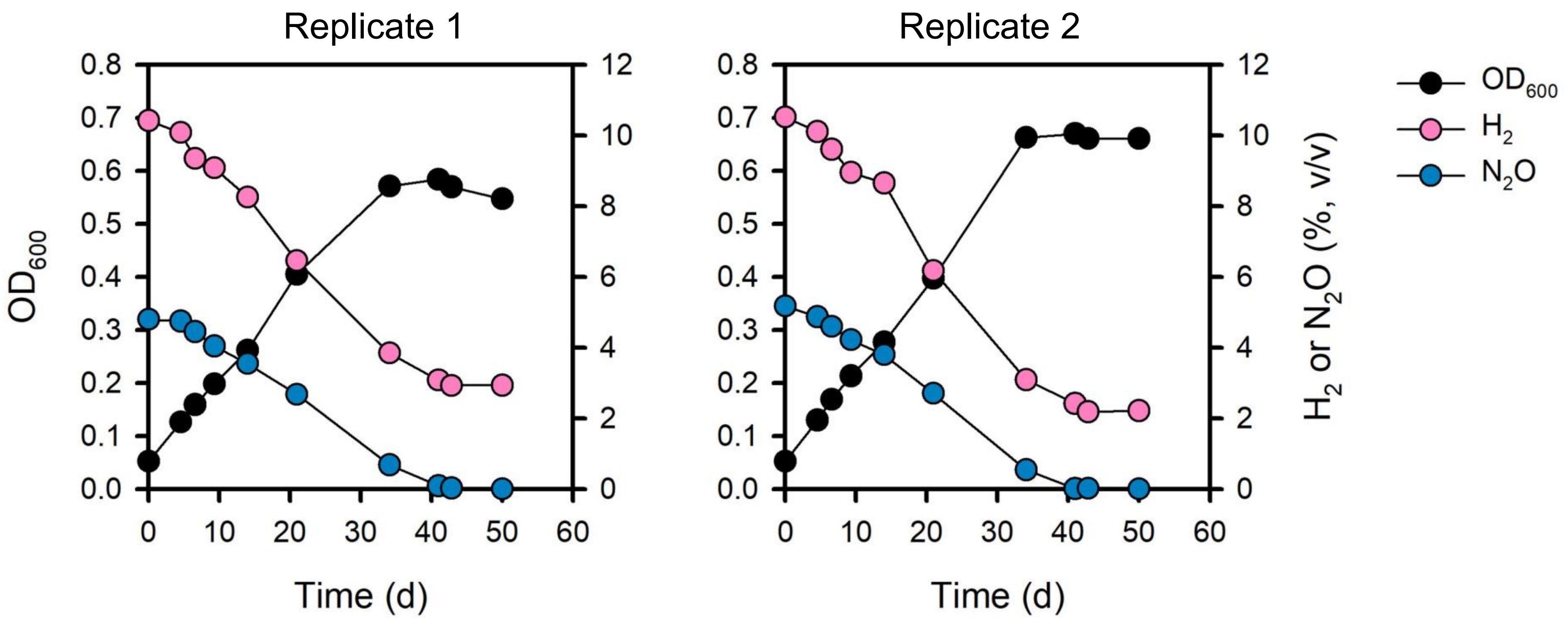

Supplementary Figure 4. Anaerobic growth of *Methylacidiphilum caldifontis* IT6 on hydrogen coupled with N_2O reduction. *Methylacidiphilum caldifontis* IT6 cells were grown in 1-liter bottles (2 replicates) containing 60 mL of LSM medium at pH 2.0 and a headspace containing 10% (v/v) H_2 as an electron donor, 5% (v/v) N_2O as an electron acceptor, and 5% (v/v) CO_2 as a carbon source. Optical density measurements at 600 nm were used to determine growth, followed by H_2 and N_2O consumption measurements in the culture bottles' headspace. Source data are provided as Source Data file.

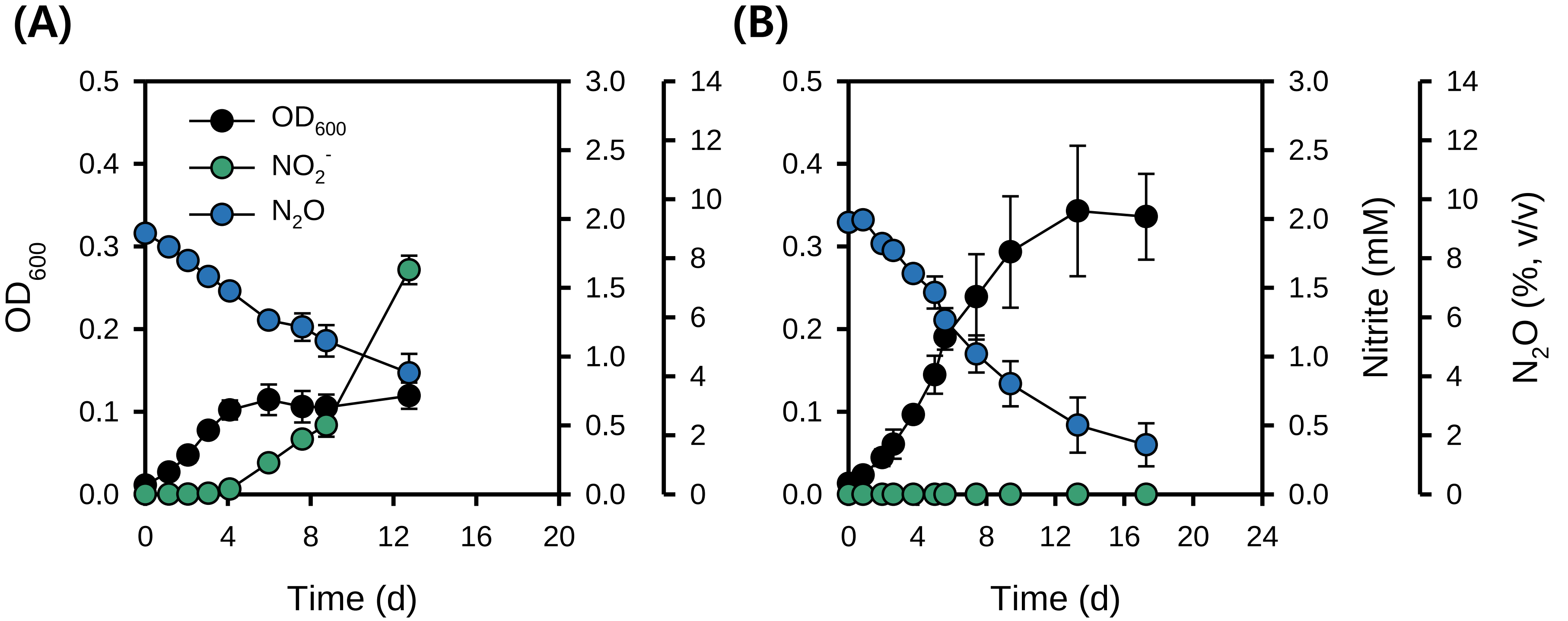

Supplementary Figure 5. The effect of NO_2^- accumulation on *Methylocella tundrae* T4 anaerobic growth on CH_3OH and N_2O. Strain T4 cells were grown anaerobically on CH_3OH and N_2O in NO_3^--containing (**A**) and NO_3^--free (**B**) media. NO_2^- formed by the reduction of NO_3^- inhibits anaerobic growth in the NO_3^--containing medium. Growth inhibition was not shown in the NO_3^--free medium. Data from **A** and **B** are the means of three biological replicates ± 1 SD. The error bars are hidden when they are smaller than symbols. Source data are provided as Source Data file.

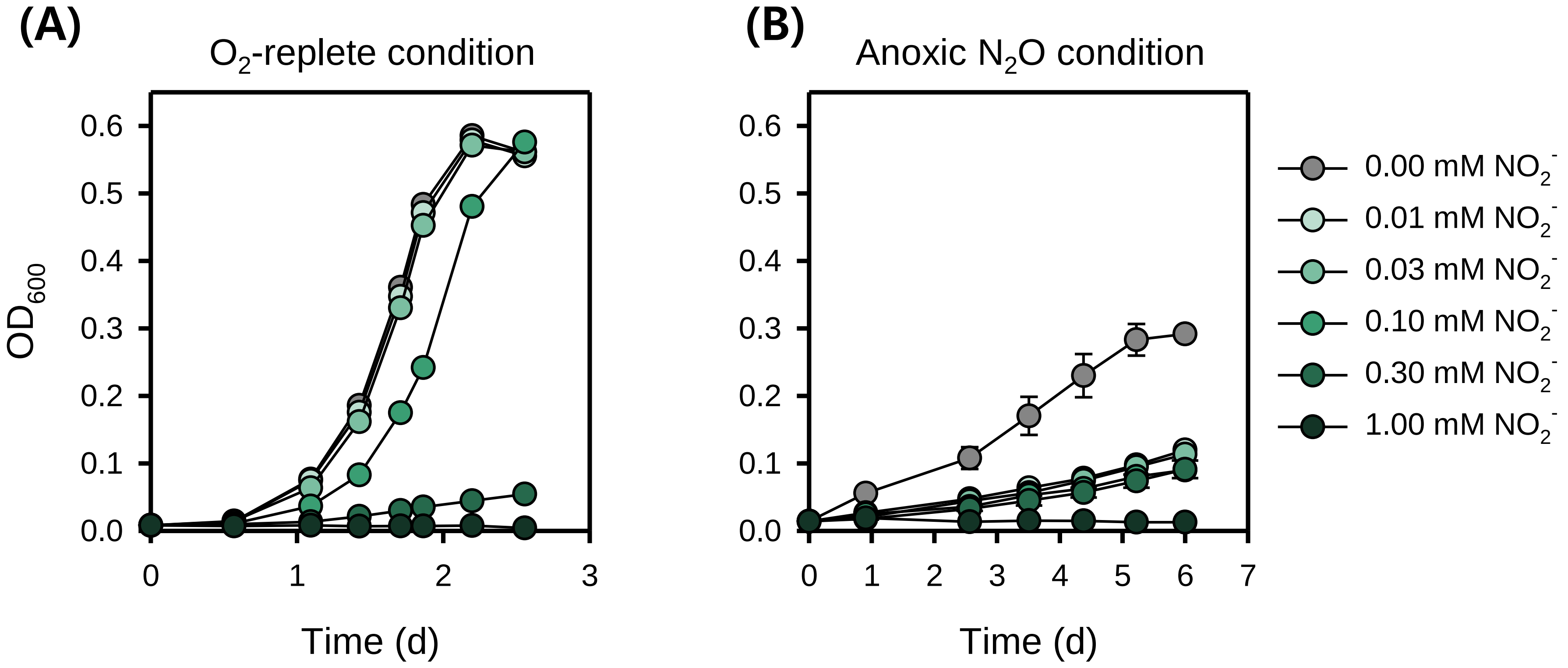

Supplementary Figure 6. Aerobic and anaerobic growth of *Methylocella tundrae* T4 on CH_3OH in response to different NO_2^- concentrations. Cultures of strain T4 grown on methanol under (**A**) O_2-replete and (**B**) anoxic N_2O-respiring growth conditions were incubated with different initial concentrations of NO_2^-. The presence of NO_2^- was inhibitory to growth in both conditions. Data from **A** and **B** are the means of three biological replicates. The error bars are hidden when they are smaller than the symbols. Source data are provided as Source Data file.

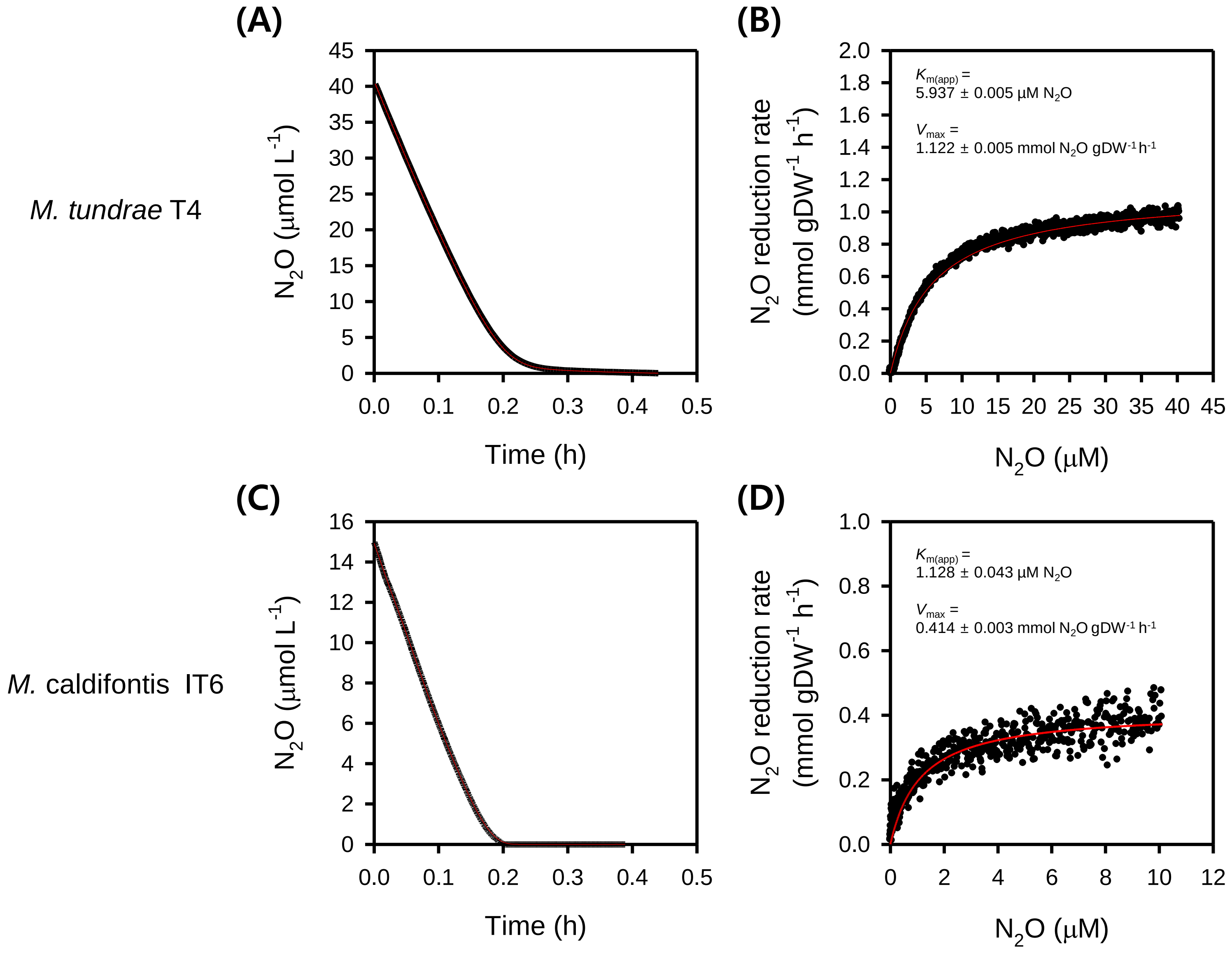
(A)
(B)
(C)
(D)
M. tundrae T4
M. caldifontis IT6
N2O (µmol L-1)
Time (h)
N2O reduction rate (mmol gDW-1 h-1)
N2O (µM)
Km(app) = 5.937 ± 0.005 µM N2O
Vmax = 1.122 ± 0.005 mmol N2O gDW-1 h-1
Km(app) = 1.128 ± 0.043 µM N2O
Vmax = 0.414 ± 0.003 mmol N2O gDW-1 h-1

Supplementary Figure 7. N_2O reduction kinetics of strains T4 and IT6. **A** N_2O reduction by anoxic CH_3OH + N_2O-grown *Methylocella tundrae* T4 cells. **B** Michaelis–Menten plot of N_2O reduction by anoxic CH_3OH + N_2O-grown T4 cells. **C** N_2O reduction by anoxic CH_3OH + N_2O-grown *Methylacidiphilum caldifontis* IT6 cells. **D** Michaelis–Menten plot of N_2O reduction by anoxic CH_3OH + N_2O-grown IT6 cells. The N_2O reduction rate was determined from microsensor measurements of methanol-dependent N_2O reduction from a single trace measurement. The Michaelis-Menten kinetic equation was fitted to the data to determine the apparent half-saturation ($K_{m(app)}$) and maximum N_2O reduction rates (V_{max}). The red line represents the best fit to the data. The standard deviations of the non-linear regression estimates are given. Source data are provided as Source Data file.

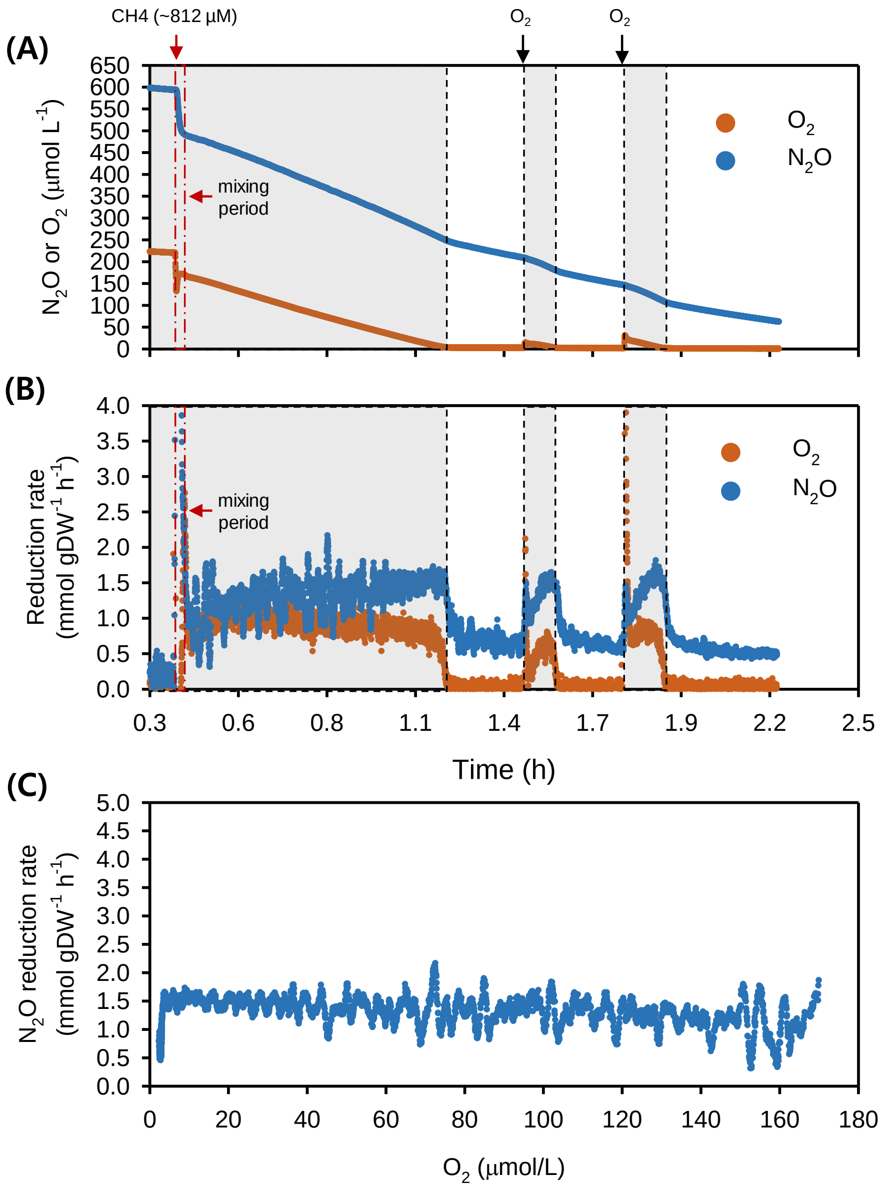

Supplementary Figure 8. CH_4-dependent N_2O reduction by *Methylocella tundrae* T4 cells at high O_2 concentrations. **A** Microrespirometry experiment demonstrating CH_4-dependent N_2O reduction at high DO concentrations by *Methylocella tundrae* T4 cells. **B** N_2O and O_2 reduction rates by cells of strain T4 during CH_4 oxidation were calculated from the upper panel (**A**). The orange and blue dots in the upper panel (**A**) represent the concentrations of dissolved O_2 and N_2O, respectively. The orange and blue dots in the bottom panel (**B**) represent the rates of O_2 and N_2O reduction, respectively. **C** N_2O reduction rates at varying O_2 concentrations are calculated from the upper panel (**A**). Experiments were performed in a microrespiration (MR) chamber fitted with O_2 and N_2O microsensors. The red and black arrows mark the addition of CH_4 (~ 812 µM) and O_2 into the MR chamber, respectively. The gray-shaded area represents periods where N_2O and O_2 are reduced simultaneously. Source data are provided as Source Data file.

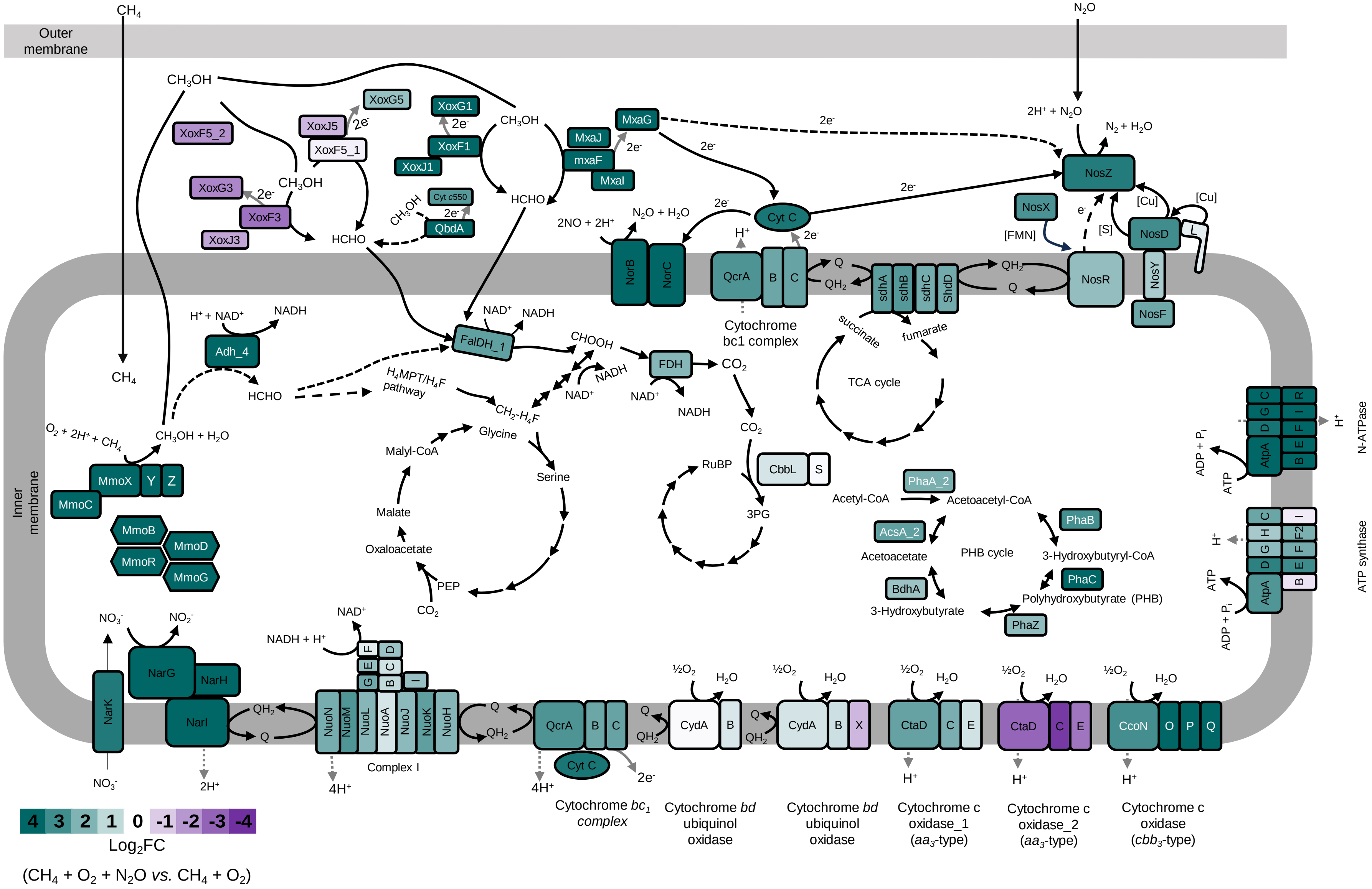

Outer membrane
Inner membrane
CH4
CH3OH
N2O
2H+ + N2O
N2 + H2O
XoxF5_2
XoxJ5
XoxF5_1
XoxG5
XoxG1
XoxF1
XoxJ1
XoxG3
XoxF3
XoxJ3
Cyt c550
QbdA
HCHO
MxaG
MxaJ
mxaF
MxaI
2e-
NosZ
NosX
[FMN]
NosR
NosD
NosL
NosY
NosF
[Cu]
[S]
e-
2NO + 2H+
N2O + H2O
NorB
NorC
Cyt C
H+
QcrA
B
C
Cytochrome bc1 complex
Q
QH2
sdhA
sdhB
sdhC
ShdD
succinate
fumarate
TCA cycle
H+ + NAD+
NADH
Adh_4
FalDH_1
NAD+
CHOOH
FDH
CO2
H4MPT/H4F pathway
CH2-H4F
Glycine
Serine
Malyl-CoA
Malate
Oxaloacetate
PEP
RuBP
3PG
CbbL
S
Acetyl-CoA
Acetoacetyl-CoA
PhaA_2
PhaB
AcsA_2
Acetoacetate
PHB cycle
3-Hydroxybutyryl-CoA
PhaC
BdhA
Polyhydroxybutyrate (PHB)
3-Hydroxybutyrate
PhaZ
O2 + 2H+ + CH4
CH3OH + H2O
MmoX
Y
Z
MmoC
MmoB
MmoD
MmoR
MmoG
ADP + Pi
ATP
AtpA
N-ATPase
ATP synthase
NO3-
NO2-
NarK
NarG
NarH
NarI
2H+
NADH + H+
NuoN
NuoM
NuoL
NuoA
NuoJ
NuoK
NuoH
E
F
D
G
I
Complex I
4H+
½O2
H2O
CydA
X
CtaD
CcoN
O
P
Q
Cytochrome bc1 complex
Cytochrome bd ubiquinol oxidase
Cytochrome c oxidase_1 (aa3-type)
Cytochrome c oxidase_2 (aa3-type)
Cytochrome c oxidase (cbb3-type)
4 3 2 1 0 -1 -2 -3 -4
Log2FC
(CH4 + O2 + N2O vs. CH4 + O2)

Supplementary Figure 9. Metabolic reconstruction and transcriptional response of methane-oxidizing *Methylocella tundrae* strain T4 cells to O_2-replete CH_4 + O_2- and suboxic CH_4 + O_2 + N_2O-growth conditions. The genes used to reconstruct the metabolic pathway are listed in Table S5. The gene products are shaded according to the relative fold change (Log_2FC) in gene expression between methane-oxidizing cells grown in suboxic CH_4 + O_2 + N_2O and O_2-replete CH_4 + O_2 conditions. Genes up-regulated in suboxic CH_4 + O_2 + N_2O-grown cells are shown in teal green, while those up-regulated in O_2-replete CH_4 + O_2-grown cells are shown in purple. Note that proteins are not drawn to scale. **Methane oxidation:** Methane is oxidized to methanol by the cytoplasmic (soluble) methane monooxygenase, sMMO (T4_01946–54). **Methanol oxidation**: Methanol is oxidized to formaldehyde in the periplasmic space by the PQQ-dependent methanol dehydrogenase (Xox- and Mxa-type), T4_03519-21, T4_00353-55, T4_01862-76. Methanol oxidation may also be mediated by the type II quinohemoprotein alcohol dehydrogenase (T4_02097-98). The $NAD(P)^+$-dependent alcohol dehydrogenase (T4_03199) may also be involved in methanol oxidation to formaldehyde in the cytoplasmic space during anaerobic growth on methanol. Formaldehyde oxidation to formate then proceeds via the tetrahydromethanopterin (H_4MPT) pathway, and C1 incorporation into the serine cycle is mediated by the tetrahydrofolate (H_4F) carbon assimilation pathway. The Calvin-Benson-Bassham pathway is also a possible route for CO_2 fixation. **Nitrous oxide reduction**: N_2O is reduced to N_2 through the activity of N_2OR in the periplasmic space. Electron transfer to NosZ occurs via cytochrome c from the cytochrome bc1 (Qcr) complex[5, 23]. Electron transfer to the NosZ may also involve direct interaction with methanol dehydrogenase c-type cytochrome (XoxG, MxaG). The NosR protein may be involved in the transfer of electrons to NosZ (refs.[5, 23]).

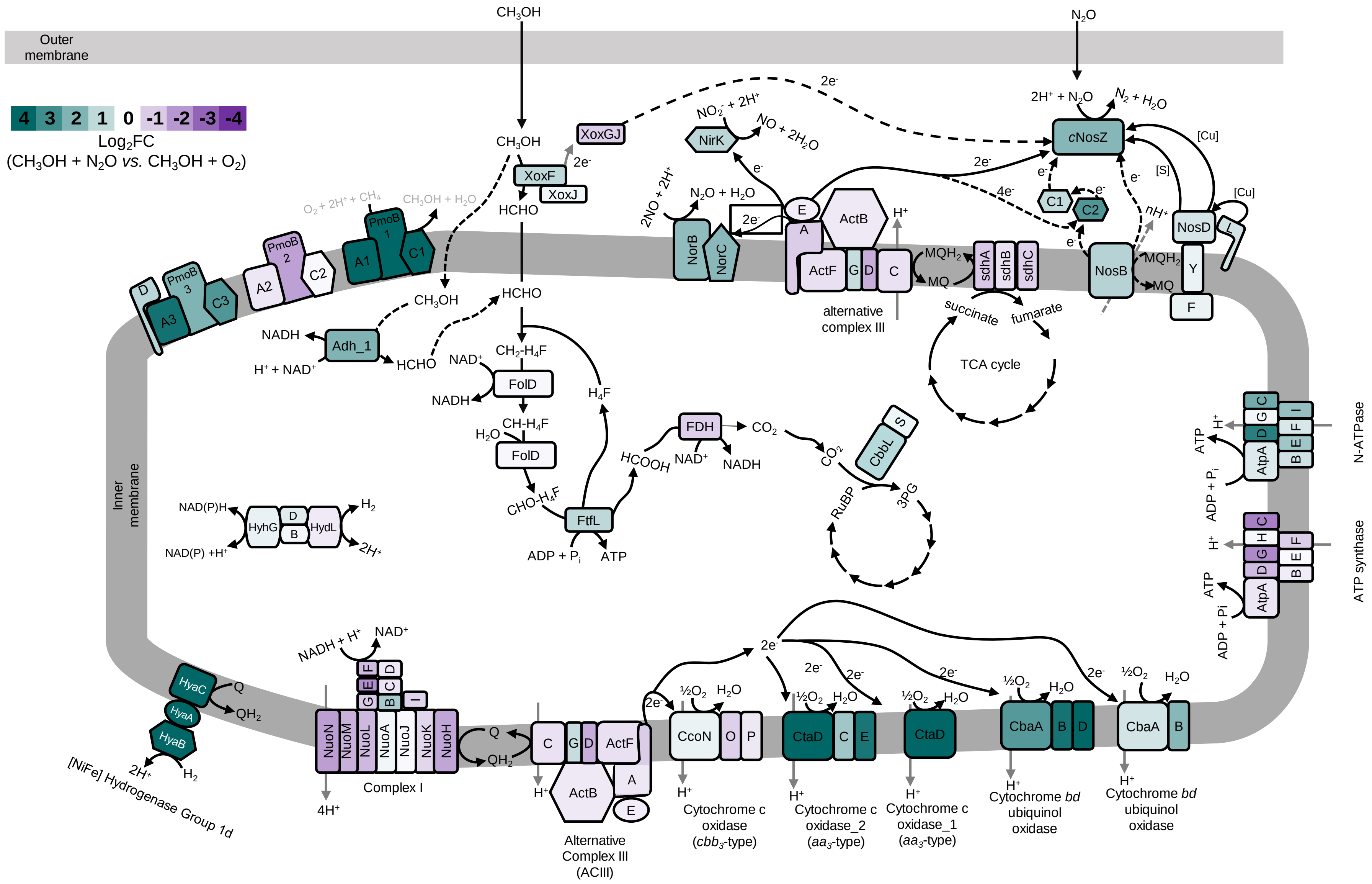

Outer membrane
Inner membrane
4 3 2 1 0 -1 -2 -3 -4
Log2FC
(CH3OH + N2O vs. CH3OH + O2)
CH3OH
N2O
XoxGJ
XoxF
XoxJ
HCHO
cNosZ
2H+ + N2O
N2 + H2O
NirK
NO + 2H2O
NorB
NorC
ActB
ActF
alternative complex III
sdhA
sdhB
sdhC
NosB
NosD
TCA cycle
succinate
fumarate
PmoB 1
PmoB 2
PmoB 3
Adh_1
NADH
FolD
FtfL
FDH
CbbL
RuBP
3PG
HyhG
HydL
AtpA
N-ATPase
ATP synthase
HyaC
HyaA
HyaB
[NiFe] Hydrogenase Group 1d
NuoN
NuoM
NuoL
NuoA
NuoJ
NuoK
NuoH
Complex I
Alternative Complex III (ACIII)
CcoN
Cytochrome c oxidase (cbb3-type)
CtaD
Cytochrome c oxidase_2 (aa3-type)
Cytochrome c oxidase_1 (aa3-type)
CbaA
Cytochrome bd ubiquinol oxidase

Supplementary Figure 10. Metabolic reconstruction and transcriptional response of methanol-oxidizing *Methylacidiphilum caldifontis* IT6 cells in O_2-replete $CH_3OH + O_2$- and anoxic $CH_3OH + N_2O$-growth conditions. The genes used to reconstruct the metabolic pathway are listed in Table S6. The gene products are shaded according to the relative fold change (Log_2FC) in gene expression between methane-oxidizing cells grown in anoxic $CH_3OH + N_2O$ and O_2-replete $CH_3OH + O_2$ conditions. Genes up-regulated in anoxic $CH_3OH + N_2O$-grown cells are shown in teal green, while those up-regulated in O_2-replete $CH_3OH + O_2$-grown cells are shown in purple. Note that proteins are not drawn to scale. **Methanol oxidation**: Methanol is oxidized to formaldehyde in the periplasmic space by the PQQ-dependent methanol dehydrogenase (Xox-type), IT6_00336-38. The $NAD(P)^+$-dependent alcohol dehydrogenase (IT6_01501 and IT6_01931) may also be involved in methanol oxidation to formaldehyde in the cytoplasmic space during anaerobic growth on methanol. Methanol oxidation produces formaldehyde, which can either spontaneously or enzymatically bind to tetrahydrofolate (H_4F) to produce methylene-tetrahydrofolate (CH_2-H_4F). FolD converts methylenetrahydrofolate (CH_2-H_4F) to methenyl-tetrahydrofolate (CH-H_4F), which is then converted to formyl-tetrahydrofolate (CHO-H_4F) by the same enzyme[13]. ATP is generated in the process of converting this product to H_4F and formate. Formate is oxidized to CO_2 through the activity of formate dehydrogenase. C1 incorporation into biomass is through the Calvin-Benson-Bassham pathway. **Nitrous oxide reduction**: Strain IT6 produces a cytochrome *c* N_2OR (*c*NosZ) with an additional C-terminal monohaem cytochrome *c* domain thought to function as an electron entry point to the active site copper. The alternative complex III (ACIII) oxidizes MQH_2 to MQ and transfers electrons to NosZ (refs.[5, 23]). The methanol dehydrogenase *c*-type cytochrome (XoxGJ) may also transfer electrons to the NosZ. Electron transfer from menaquinol to NosZ is predicted to involve NosB, NosC1, and NosC2 (refs.[5, 23, 24]).

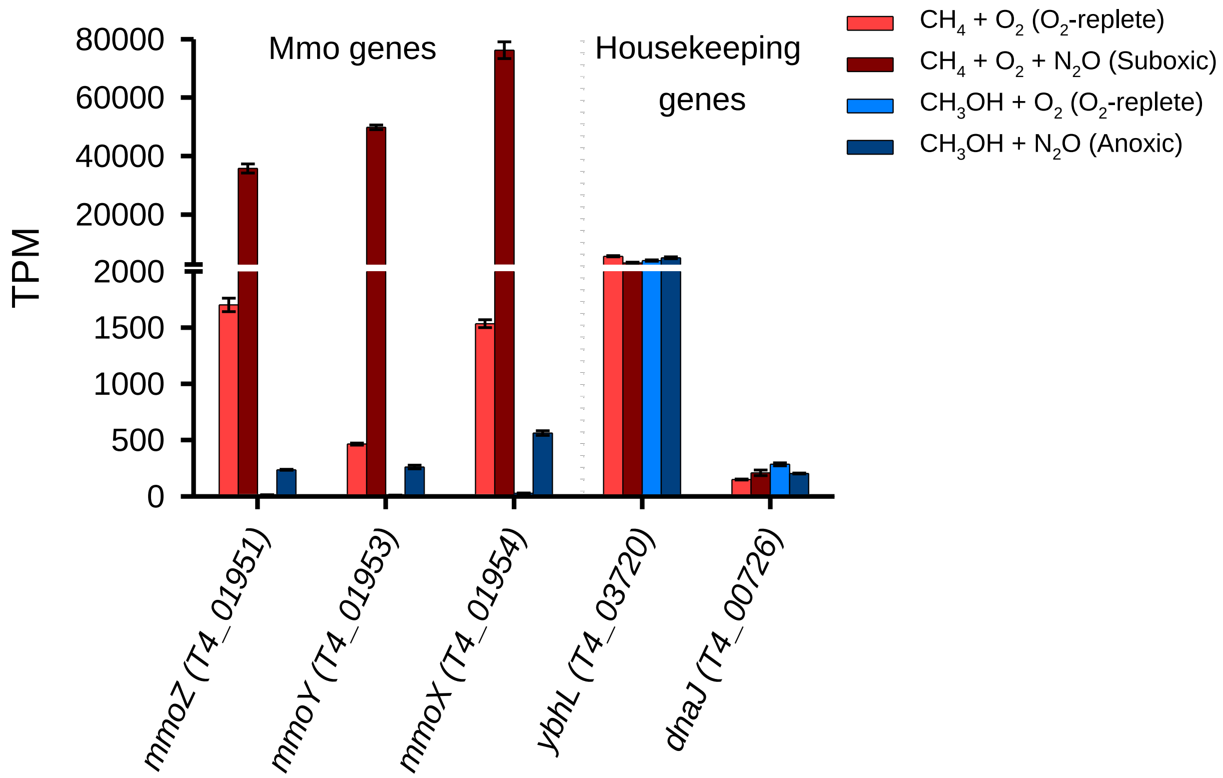

Supplementary Figure 11. Expression patterns of genes encoding methane monooxygenase in *Methylocella tundrae* T4. Cells grown under O_2-replete ($CH_4 + O_2$ and $CH_3OH + O_2$), suboxic ($CH_4 + O_2 + N_2O$), and anoxic ($CH_3OH + N_2O$) growth conditions were analyzed. Each bar represents gene expression levels in transcripts per kilobase million (TPM) for cells grown under various conditions, as indicated by the color bars. Expression levels of the housekeeping genes, *ybhL* and *dnaJ*, are shown for comparison. Error bars represent ±1 SD for four biological replicates of each set. Source data are provided as Source Data file.

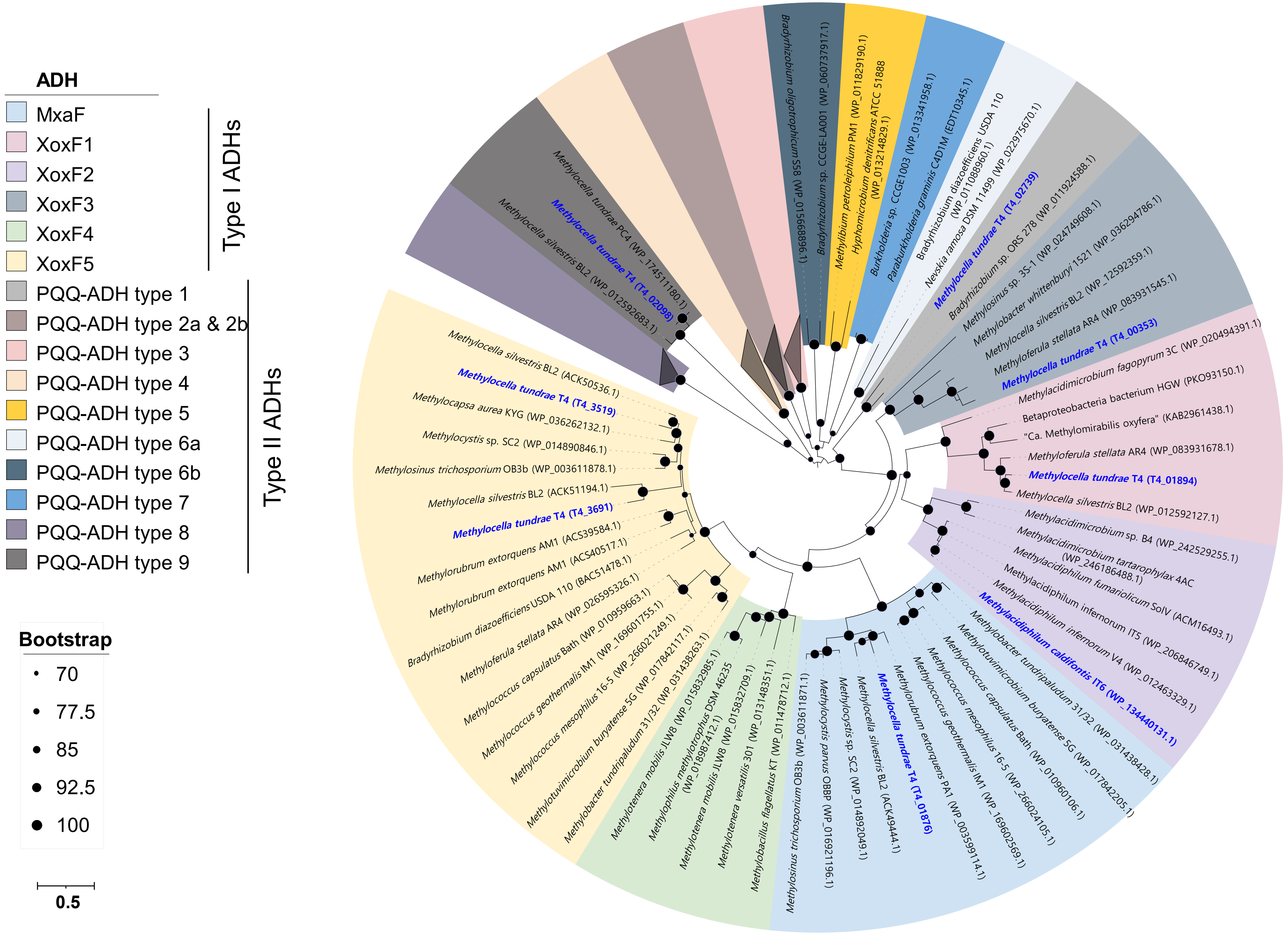

ADH
MxaF
XoxF1
XoxF2
XoxF3
XoxF4
XoxF5
PQQ-ADH type 1
PQQ-ADH type 2a & 2b
PQQ-ADH type 3
PQQ-ADH type 4
PQQ-ADH type 5
PQQ-ADH type 6a
PQQ-ADH type 6b
PQQ-ADH type 7
PQQ-ADH type 8
PQQ-ADH type 9
Type I ADHs
Type II ADHs
Bootstrap
70
77.5
85
92.5
100
0.5
Bradyrhizobium oligotrophicum S58 (WP_015668896.1)
Bradyrhizobium sp. CCGE-LA001 (WP_060737917.1)
Methylibium petroleiphilum PM1 (WP_011829190.1)
Hyphomicrobium denitrificans ATCC 51888 (WP_013214829.1)
Burkholderia sp. CCGE1003 (WP_013341958.1)
Paraburkholderia graminis C4D1M (EDT10345.1)
Bradyrhizobium diazoefficiens USDA 110 (WP_011088960.1)
Nevskia ramosa DSM 11499 (WP_022975670.1)
Methylocella tundrae T4 (T4_02739)
Bradyrhizobium sp. ORS 278 (WP_011924588.1)
Methylosinus sp. 3S-1 (WP_024749608.1)
Methylobacter whittenburyi 1521 (WP_036294786.1)
Methylocella silvestris BL2 (WP_12592359.1)
Methyloferula stellata AR4 (WP_083931545.1)
Methylocella tundrae T4 (T4_00353)
Methylacidimicrobium fagopyrum 3C (WP_020494391.1)
Betaproteobacteria bacterium HGW (PKO93150.1)
"Ca. Methylomirabilis oxyfera" (KAB2961438.1)
Methyloferula stellata AR4 (WP_083931678.1)
Methylocella tundrae T4 (T4_01894)
Methylocella silvestris BL2 (WP_012592127.1)
Methylacidimicrobium sp. B4 (WP_242529255.1)
Methylacidimicrobium tartarophylax 4AC (WP_246186488.1)
Methylacidiphilum fumariolicum SolV (ACM16493.1)
Methylacidiphilum infernorum ITS (WP_206846749.1)
Methylacidiphilum infernorum V4 (WP_012463329.1)
Methylacidiphilum caldifontis IT6 (WP_134440131.1)
Methylobacter tundripaludum 31/32 (WP_031438428.1)
Methylotuvimicrobium buryatense 5G (WP_017842205.1)
Methylococcus capsulatus Bath (WP_010960106.1)
Methylococcus mesophilus 16-5 (WP_266024105.1)
Methylococcus geothermalis IM1 (WP_169602569.1)
Methylorubrum extorquens PA1 (WP_003599114.1)
Methylocella tundrae T4 (T4_01876)
Methylocella silvestris BL2 (ACK49444.1)
Methylocystis sp. SC2 (WP_014892049.1)
Methylocystis parvus OBBP (WP_016921196.1)
Methylosinus trichosporium OB3b (WP_003611871.1)
Methylobacillus flagellatus KT (WP_011478712.1)
Methylotenera versatilis 301 (WP_013148351.1)
Methylotenera mobilis JLW8 (WP_015832709.1)
Methylophilus methylotrophus DSM 46235 (WP_018987412.1)
Methylotenera mobilis JLW8 (WP_015832985.1)
Methylobacter tundripaludum 31/32 (WP_031438263.1)
Methylotuvimicrobium buryatense 5G (WP_017842117.1)
Methylococcus mesophilus 16-5 (WP_266021249.1)
Methylococcus geothermalis IM1 (WP_169601755.1)
Methylococcus capsulatus Bath (WP_010959663.1)
Methyloferula stellata AR4 (WP_026595326.1)
Bradyrhizobium diazoefficiens USDA 110 (BAC51478.1)
Methylorubrum extorquens AM1 (ACS40517.1)
Methylorubrum extorquens AM1 (ACS39584.1)
Methylocella tundrae T4 (T4_3691)
Methylocella silvestris BL2 (ACK51194.1)
Methylosinus trichosporium OB3b (WP_003611878.1)
Methylocystis sp. SC2 (WP_014890846.1)
Methylocapsa aurea KYG (WP_036262132.1)
Methylocella tundrae T4 (T4_3519)
Methylocella silvestris BL2 (ACK50536.1)
Methylocella silvestris BL2 (WP_012592683.1)
Methylocella tundrae T4 (T4_02098)
Methylocella tundrae PC4 (WP_174511180.1)

Supplementary Figure 12. Phylogenetic analysis of PQQ-dependent alcohol dehydrogenases (type I quinoproteins and type II quinohemoprotein). Different subclasses or clades of the type I quinoproteins and type II quinohemoprotein are shown. A maximum-likelihood tree was inferred with IQ-TREE (IQ-TREE options: -B 1000 -m LG+F+R5 -T AUTO) and rooted at the mid-point. Bootstrap values ≥ 70% based on 1,000 replications are indicated. PQQ-dependent ADH of strains T4 and IT6 are labeled blue.

Supplementary References

1. Yoon S, Nissen S, Park D, Sanford RA, Löffler FE. Nitrous oxide reduction kinetics distinguish bacteria harboring clade I NosZ from those harboring clade II NosZ. *Appl Environ Microbiol* **82**, 3793-3800 (2016).

2. Suenaga T, Riya S, Hosomi M, Terada A. Biokinetic characterization and activities of N_2O-reducing bacteria in response to various oxygen levels. *Front Microbiol* **9**, 697 (2018).

3. Bergaust L, van Spanning RJM, Frostegård Å, Bakken LR. Expression of nitrous oxide reductase in *Paracoccus denitrificans* is regulated by oxygen and nitric oxide through FnrP and NNR. *Microbiology (Reading, England)* **158**, 826-834 (2012).

4. Spiro S. Nitrous oxide production and consumption: regulation of gene expression by gas-sensitive transcription factors. *Philos Trans R Soc Lond B Biol Sci* **367**, 1213-1225 (2012).

5. Torres MJ, et al. Nitrous oxide metabolism in nitrate-reducing bacteria: physiology and regulatory mechanisms. *Adv Microb Physiol* **68**, 353-432 (2016).

6. Kern M, Simon J. Three transcription regulators of the Nss family mediate the adaptive response induced by nitrate, nitric oxide or nitrous oxide in *Wolinella succinogenes*. *Environ Microbiol* **18**, 2899-2912 (2016).

7. Kellermann R, Hauge K, Tjåland R, Thalmann S, Bakken LR, Bergaust L. Preparation for denitrification and phenotypic diversification at the cusp of anoxia: a purpose for N_2O reductase vis-à-vis multiple roles of O_2. *Appl Environ Microbiol* **88**, e0105322 (2022).

8. Van Spanning RJ, De Boer AP, Reijnders WN, Westerhoff HV, Stouthamer AH, Van Der Oost J. FnrP and NNR of *Paracoccus denitrificans* are both members of the FNR family of transcriptional activators but have distinct roles in respiratory adaptation in response to oxygen limitation. *Mol Microbiol* **23**, 893-907 (1997).

9. Durand S, Guillier M. Transcriptional and post-transcriptional control of the nitrate respiration in bacteria. *Front Mol Biosci* **8**, 667758 (2021).

10. Morvan C, Folgosa F, Kint N, Teixeira M, Martin-Verstraete I. Responses of *Clostridia* to oxygen: from detoxification to adaptive strategies. *Environ Microbiol* **23**, 4112-4125 (2021).

11. Bouchal P, et al. Unraveling an FNR based regulatory circuit in *Paracoccus denitrificans* using a proteomics-based approach. *Biochim Biophys Acta Proteins Proteomics* **1804**, 1350-1358 (2010).

12. Degli Esposti M, Mentel M, Martin W, Sousa FL. Oxygen reductases in alphaproteobacterial genomes: physiological evolution from low to high oxygen environments. *Front Microbiol* **10**, 499 (2019).

13. Schmitz RA, et al. Verrucomicrobial methanotrophs: ecophysiology of metabolically versatile acidophiles. *FEMS Microbiol Rev* **45**, fuab007 (2021).

14. García-Horsman JA, Barquera B, Rumbley J, Ma J, Gennis RB. The superfamily of heme-copper respiratory oxidases. *J Bacteriol* **176**, 5587-5600 (1994).

15. D'Mello R, Hill S, Poole RK. The cytochrome bd quinol oxidase in *Escherichia coli* has an extremely high oxygen affinity and two oxygen-binding haems: implications for regulation of activity in vivo by oxygen inhibition. *Microbiology (Reading, England)* **142 (Pt 4)**, 755-763 (1996).

16. Hirai T, Osamura T, Ishii M, Arai H. Expression of multiple *cbb*$_3$ cytochrome c oxidase isoforms by combinations of multiple isosubunits in *Pseudomonas aeruginosa*. *P Natl Acad Sci USA* **113**, 12815-12819 (2016).

17. Hirayama H, et al. Multispecies populations of methanotrophic *Methyloprofundus* and cultivation of a Likely dominant species from the Iheya North Deep-Sea hydrothermal field. *Appl Environ Microbiol* **88**, e0075821 (2022).

18. Trojan D, et al. Microaerobic lifestyle at nanomolar O_2 concentrations mediated by low-affinity terminal oxidases in abundant soil bacteria. *mSystems* **6**, e0025021 (2021).

19. Sedlacek CJ, et al. Transcriptomic response of Nitrosomonas europaea transitioned from ammonia- to oxygen-limited steady-state growth. *mSystems* **5**, e00562-00519 (2020).

20. Rauhamäki V, Wikström M. The causes of reduced proton-pumping efficiency in type B and C respiratory heme-copper oxidases, and in some mutated variants of type A. *Biochim Biophys Acta Bioenerg* **1837**, 999-1003 (2014).

21. Buschmann S, Warkentin E, Xie H, Langer JD, Ermler U, Michel H. The structure of cbb3 cytochrome oxidase provides insights into proton pumping. *Science* **329**, 327-330 (2010).

22. Iwata S, Ostermeier C, Ludwig B, Michel H. Structure at 2.8 Å resolution of cytochrome c oxidase from *Paracoccus denitrificans*. *Nature* **376**, 660-669 (1995).

23. Hein S, Simon J. Bacterial nitrous oxide respiration: electron transport chains and copper transfer reactions. *Adv Microb Physiol* **75**, 137-175 (2019).

24. Simon J, Einsle O, Kroneck PM, Zumft WG. The unprecedented nos gene cluster of *Wolinella succinogenes* encodes a novel respiratory electron transfer pathway to cytochrome c nitrous oxide reductase. *FEBS Lett* **569**, 7-12 (2004).
