# Supplementary material for: Nitrous oxide respiration in acidophilic methanotrophs
Source: Nat Commun. 2024 May 18;15:4226. doi: 10.1038/s41467-024-48161-z (PMC11102522; doi:10.1038/s41467-024-48161-z)
Supplement: Supplementary file 3 — Description of Additional Supplementary Files [file 41467_2024_48161_MOESM3_ESM.pdf]

Description of Additional Supplementary Files

Supplementary Dataset 1: Distribution of denitrification genes in isolates and metagenome-assembled genomes of methanotrophs. Genes encoding the soluble and particulate methane monooxygenases were included. The presence of each gene in target genomes is denoted by the green color bar and the number of copies is included within the bar. Published genomes and MAGs were compiled from the NCBI database.

Supplementary Dataset 2: The *nos* gene cluster (NGC) of methanotrophs. The highest percent similarity with the translated amino acid sequence of the genes of NGC and entries in the NCBI database is shown.

Supplementary Dataset 3: Genomic island-related genes found in the genome of *Methylacidiphilum caldifontis* IT6. The genomic islands were identified with IslandViewer 4.

Supplementary Dataset 4: N_2O-dependent anaerobic growth of N_2OR-containing and N_2OR-lacking strains of genera *Methylocella* and *Methylacidiphilum* under different electron donors. Growth is indicated as: "+" if the increase in OD_{600} > 0.02; "−" if the increase in OD_{600} < 0.005; "ND", not determined. All cultures were cultivated in LSM media at pH 2 (*Methylacidiphilum* spp.) and pH 5.5 (*Methylocella* spp.).

Supplementary Dataset 5: Central metabolic genes in *Methylocella tundrae* T4 and their differential expression under methanol- (CH_3OH + N_2O versus CH_3OH + O_2) and methane- (CH_4 + O_2 + N_2O versus CH_4 + O_2) oxidizing growth conditions. Genes were considered upregulated if the Log_2FC was higher than [0.85] or downregulated if lower than [-1.0] with an adjusted *p*-value ≤ 0.05. Data are from four biological replicates. For easy comparisons between samples, TPM (Transcripts Per Kilobase Million) values were calculated. The p-values were calculated using a two-sided Wald test, and multiple-comparison adjustments were made using the Benjamini-Hochberg method to obtain the adjusted p-values in DESeq2.

Supplementary Dataset 6: Transcriptional regulation of selected key genes in *Methylocella tundrae* T4 cells grown under methanol-oxidizing (anoxic CH_3OH + N_2O versus O_2-replete CH_3OH + O_2) and methane-oxidizing (suboxic CH_4 + O_2 + N_2O versus O_2-replete CH_4 + O_2) growth conditions. Differences in expression were considered upregulated if the Log_2FC was higher than [0.85] or downregulated if lower than [-1.0] with an adjusted *p*-value ≤ 0.05. Data from four biological replicates. The *p*-values were calculated using a two-sided Wald test, and multiple-comparison adjustments were made using the Benjamini-Hochberg method to obtain the adjusted p-values in DESeq2.

Supplementary Dataset 7: Central metabolic genes in *Methylacidiphilum caldifontis* IT6 and their differential expression under anoxic CH_3OH + N_2O *vs.* O_2-replete CH_3OH + O_2-growth conditions. Genes were considered upregulated if the Log_2FC was higher than [0.85] or downregulated if lower than [-1.0] with an adjusted p-value ≤ 0.05. Data are from four or five biological replicates. For easy comparisons between samples, TPM (Transcripts Per Kilobase Million) values were calculated. The p-

values were calculated using a two-sided Wald test, and multiple-comparison adjustments were made using the Benjamini-Hochberg method to obtain the adjusted p-values in DESeq2.
