# Supplementary material for: Nitrous oxide respiration in acidophilic methanotrophs
Source: Nat Commun. 2024 May 18;15:4226. doi: 10.1038/s41467-024-48161-z (PMC11102522; doi:10.1038/s41467-024-48161-z)
Supplement: Supplementary file 5 — Reporting Summary [file 41467_2024_48161_MOESM5_ESM.pdf]

nature portfolio

Corresponding author(s): Sung-Keun Rhee

Last updated by author(s): Apr 4, 2024

Reporting Summary

Nature Portfolio wishes to improve the reproducibility of the work that we publish. This form provides structure for consistency and transparency in reporting. For further information on Nature Portfolio policies, see our Editorial Policies and the Editorial Policy Checklist.

Statistics

For all statistical analyses, confirm that the following items are present in the figure legend, table legend, main text, or Methods section.

n/a	Confirmed	
[ ]	[x]	The exact sample size (*n*) for each experimental group/condition, given as a discrete number and unit of measurement
[ ]	[x]	A statement on whether measurements were taken from distinct samples or whether the same sample was measured repeatedly
[ ]	[x]	The statistical test(s) used AND whether they are one- or two-sided<br>*Only common tests should be described solely by name; describe more complex techniques in the Methods section.*
[x]	[ ]	A description of all covariates tested
[x]	[ ]	A description of any assumptions or corrections, such as tests of normality and adjustment for multiple comparisons
[ ]	[x]	A full description of the statistical parameters including central tendency (e.g. means) or other basic estimates (e.g. regression coefficient) AND variation (e.g. standard deviation) or associated estimates of uncertainty (e.g. confidence intervals)
[ ]	[x]	For null hypothesis testing, the test statistic (e.g. *F*, *t*, *r*) with confidence intervals, effect sizes, degrees of freedom and *P* value noted<br>*Give P values as exact values whenever suitable.*
[x]	[ ]	For Bayesian analysis, information on the choice of priors and Markov chain Monte Carlo settings
[x]	[ ]	For hierarchical and complex designs, identification of the appropriate level for tests and full reporting of outcomes
[x]	[ ]	Estimates of effect sizes (e.g. Cohen's *d*, Pearson's *r*), indicating how they were calculated

Our web collection on statistics for biologists contains articles on many of the points above.

Software and code

Policy information about availability of computer code

Data collection	Illumina HiSeq4000, PacBio RS II platforms were used to collect reads of genome. MinKNOW version 23.04.6 was used for collecting Oxford Nanopore Technologies subreads. Illumina NovaSeq6000 was used to collect reads of transcriptome. All data from the microsensor multimeter was logged onto a laptop using SensorTrace Suite software (v.3.3.0; Unisense). Gas chromatograph data were collected with the Agilent OpenLab CDS ChemStation (v2.3.54).
Data analysis	The data was analyzed without the use of any custom algorithms or software. All algorithms and software used are freely available in open-source packages. They software tools used in data analysis of this present study are listed as follows:<br><br>Trycycler (v0.5.4), CheckM (v1.2.2), Miniasm/Minpolish (v0.3-r179), Flye (v2.9.3), Raven (v1.8.3), Polypolish (v0.5.0), POLCA (v4.0.5), SignalP (v6.0), TMHMM (v2.0), InterPro v94.0, GO, PFAM (v35.0), CDD (v3.20), TIGRFAM (v.15.0), EggNOG (v.5.0), Flye (v2.9.2), Prokka (v1.14.6), PGAP (v4.2), MAFFT (v7.511), IQ-TREE (v1.6.12), iTOL (v.6.7.2), IslandViewer4, HTSeq (v0.12.3), R (v4.3.2), DESeq2 (1.40.2), FastQC (v0.11.8), Trimmomatic (v0.36), SortMeRNA (v4.3.6), Bowtie2 (v2.4.4), SigmaPlot (v10.0).

For manuscripts utilizing custom algorithms or software that are central to the research but not yet described in published literature, software must be made available to editors and reviewers. We strongly encourage code deposition in a community repository (e.g. GitHub). See the Nature Portfolio guidelines for submitting code & software for further information.

Data

Policy information about availability of data

All manuscripts must include a data availability statement. This statement should provide the following information, where applicable:

- Accession codes, unique identifiers, or web links for publicly available datasets
- A description of any restrictions on data availability
- For clinical datasets or third party data, please ensure that the statement adheres to our policy

All numerical data used to make the figures is provided in source data. The complete genome sequence of strain T4 was deposited in the National Center for Biotechnology Information (NCBI) GenBank (accession nos. CP139089 [https://www.ncbi.nlm.nih.gov/nuccore/CP139089/] (Chromosome), CP139088 [https://www.ncbi.nlm.nih.gov/nuccore/CP139088/] (Plasmid 1), and CP139087 [https://www.ncbi.nlm.nih.gov/nuccore/CP139087/] (Plasmid 2)). The genomic sequences and genome annotations of Methylocystis species (strains IM2, IM3, and IM4) and 'Ca. Methylotropicum kingii' are available on Figshare (https://doi.org/10.6084/m9.figshare.25521913.v2). All previously sequenced genomes analyzed in this study are available in the NCBI Database with the GenBank accession numbers listed in Supplementary Dataset 1. The whole transcriptome data was deposited in the NCBI BioProject database under the accession number PRJNA1050235 [https://www.ncbi.nlm.nih.gov/bioproject/PRJNA1050235]. The following are the publicly available databases/datasets used in the study: NCBI NR [https://www.ncbi.nlm.nih.gov/refseq/], BV-BRC [https://www.bv-brc.org/], NCyc [https://github.com/qichao1984/NCyc], Pfam [https://pfam.xfam.org/], InterPro [https://www.ebi.ac.uk/interpro/], GO [https://geneontology.org/], CDD [https://www.ncbi.nlm.nih.gov/cdd/], TIGRFAM [https://tigrfams.jcvi.org/cgi-bin/index.cgi], and EggNOG [https://tigrfams.jcvi.org/cgi-bin/index.cgi]. Source data are provided with this paper.

Research involving human participants, their data, or biological material

Policy information about studies with human participants or human data. See also policy information about sex, gender (identity/presentation), and sexual orientation and race, ethnicity and racism.

Reporting on sex and gender	n/a
Reporting on race, ethnicity, or other socially relevant groupings	n/a
Population characteristics	n/a
Recruitment	n/a
Ethics oversight	n/a

Note that full information on the approval of the study protocol must also be provided in the manuscript.

Field-specific reporting

Please select the one below that is the best fit for your research. If you are not sure, read the appropriate sections before making your selection.

☒ Life sciences ☐ Behavioural & social sciences ☐ Ecological, evolutionary & environmental sciences

For a reference copy of the document with all sections, see nature.com/documents/nr-reporting-summary-flat.pdf

Life sciences study design

All studies must disclose on these points even when the disclosure is negative.

Sample size	No statistical methods were used to estimate the sample size. Our goal in selecting this sample size was to ensure reproducibility in our experiments and that we could statistically analyze gene expression profiles across all growth conditions. The method section specifies all sample sizes that were used. Experiments measuring growth typically used a sample size of n = 3, while transcriptome experiments typically used n = 4.
Data exclusions	No data was excluded
Replication	All physiological experiments were repeated >=3 times (>=3 biological replicates). Transcriptome experiments were performed 4 to 5 times. All replication attempts were successful, and all biological replicates are presented in the manuscript.
Randomization	Randomization was not applicable in the study. We used the same pure cultures of methanotrophs (Methylocella tundrae T4, Methylocella silvestris BL2, Methylacidiphilum caldifontis IT6, Methylacidiphilum infernorum IT5, Methylocystis sp. SC2, Methylocystis spp. (strains IM1, IM3, and IM4) for all experiments.
Blinding	Binding is not relevant in this study since we used pure cultures of methanotrophs in all experiments.

Reporting for specific materials, systems and methods

We require information from authors about some types of materials, experimental systems and methods used in many studies. Here, indicate whether each material, system or method listed is relevant to your study. If you are not sure if a list item applies to your research, read the appropriate section before selecting a response.

Materials & experimental systems

n/a	Involved in the study
☒	☐ Antibodies
☒	☐ Eukaryotic cell lines
☒	☐ Palaeontology and archaeology
☒	☐ Animals and other organisms
☒	☐ Clinical data
☒	☐ Dual use research of concern
☒	☐ Plants

Methods

n/a	Involved in the study
☒	☐ ChIP-seq
☒	☐ Flow cytometry
☒	☐ MRI-based neuroimaging

Plants

Seed stocks	n/a
Novel plant genotypes	n/a
Authentication	n/a
